# Crystal Growth Kinetics of Na_**2**_CO_**3**_ Hydrate Phases in the Na_**2**_CO_**3**_–NaOH–H_**2**_O System for Sustainable Soda Ash Production

**DOI:** 10.1021/acsomega.5c09440

**Published:** 2026-01-23

**Authors:** Somayyeh Ghaffari, Jonathan Gänsch, Peter Schulze, Heike Lorenz

**Affiliations:** 28307 Max Planck Institute for Dynamics of Complex Technical Systems, Magdeburg 39106 Germany

## Abstract

As part of the CODA project, which is laying the foundation for a novel sustainable soda ash (Na_2_CO_3_) production strategy, this study provides essential parameters and insights for the design, optimization, and scale-up of the required crystallization processes. It investigates the crystal growth kinetics of Na_2_CO_3_·10H_2_O and Na_2_CO_3_·1H_2_O within the Na_2_CO_3_–NaOH–H_2_O system using cooling and vacuum evaporative crystallization, respectively. Experimental studies were conducted utilizing a shortcut method based on seeded batch experiments in a 3 L-scale. The growth rate of Na_2_CO_3_·10H_2_O was studied over a temperature range of 17–6 °C, while Na_2_CO_3_·1H_2_O was examined at a constant temperature of 50 °C under a fixed evaporation rate. Minimal secondary nucleation was confirmed through offline crystal size distribution measurements. Na_2_CO_3_·10H_2_O growth peaked at a critical supersaturation (∼1.03) and afterward slowed despite further increases in supersaturation, alluding to a potential crystal surface roughening mechanism. The presence of NaOH did not alter the crystal habit of Na_2_CO_3_·10H_2_O but did alter that of Na_2_CO_3_·1H_2_O. Despite the relatively low supersaturation, the monohydrate exhibited a higher growth rate than the decahydrate. Furthermore, the monohydrate produced yielded dense (heavy) soda ash, addressing a significant challenge within the soda ash industry.

## Graphical abstract

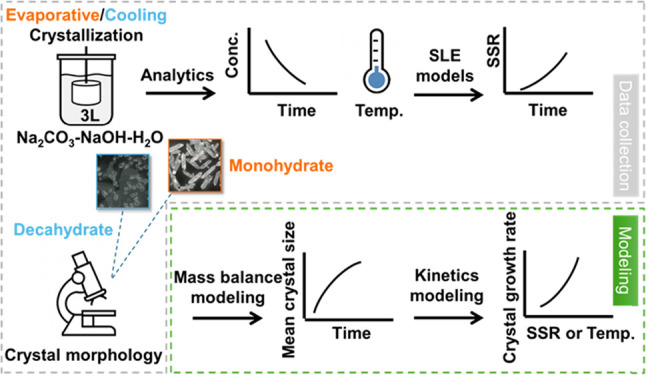

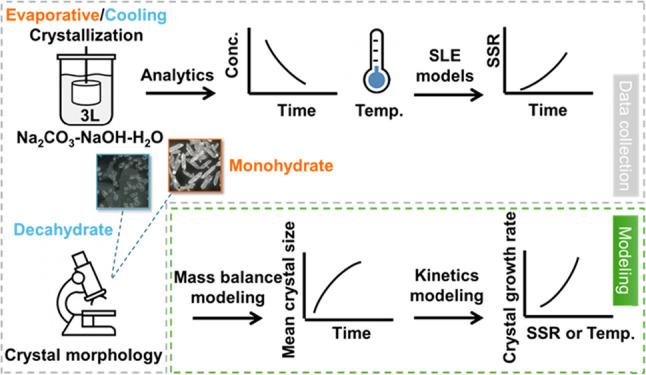

## 1 Introduction

Sodium carbonate (Na_2_CO_3_), or soda ash, is a key industrial chemical with a global production of 65 million metric tons in 2023, including both natural and synthetic sources.^1^ It is marketed as light and heavy soda ash, with the latter offering higher bulk density (850–1100 kg/m^3^) and improved transport efficiency.^2^ Primary production methods include the “monohydrate process” using trona ore and the synthetic Solvay process.

The Monohydrate process is commonly used to produce Na_2_CO_3_ from trona ore.^3^ The Monohydrate process in Wyoming is shown in Supporting Information (SI) and Figure S1 (left). It involves calcination of the trona in rotary kilns, dissolution, purification, and evaporative crystallization of Na_2_CO_3_·1H_2_O, followed by drying to yield dense anhydrous Na_2_CO_3_. Crystallization is essential, as direct calcination results in low bulk density (600–800 kg/m^3^), while the process increases it to ∼1000 kg/m^3^. The decomposition of sodium bicarbonate (NaHCO_3_) is the most energy-intensive step, requiring high-pressure steam.^4^

Where natural sources are unavailable, the Solvay process is used.^5^ As illustrated in the SI, Figure S1 (right), it involves reacting NaCl brine with CO_2_ and NH_3_ to form NaHCO_3_, which is calcined to Na_2_CO_3_. CO_2_ is provided from CaCO_3_ decomposition using coke. Limestone decomposition also produces CaO, forming Ca­(OH)_2_ to regenerate NH_3_. Though ammonia is largely recycled, losses must be replenished. Despite the Solvay’s process’s cyclic nature, the process faces challenges such as high energy use from calcination, CaCl_2_ byproduct disposal, and emissions of 790–1160 kg CO_2_ per ton of Na_2_CO_3_,^6^ prompting the need for more sustainable alternatives.

An innovative alternative to the traditional Solvay process is the recently developed CODA (Carbon-negative sODA ash) process, which offers a more sustainable approach by using CO_2_ from direct air capture and NaOH from chlor-alkali electrolysis. The overall reaction is given as Rxn [Disp-formula eq1] below.^7^ Operated under milder conditions below 150 °C, CODA involves reactive CO_2_ absorption to form carbonate ions, followed by the crystallization of hydrated Na_2_CO_3_ and drying to produce soda ash. With renewable energy, it can avoid up to 790 kg of CO_2_ emissions and capture an additional amount of 60–170 kg of CO_2_ per ton of soda ash (depending on the process variant). In total, this results in 850–960 kg less of CO_2_ emitted compared to the Solvay process.^6^

Rxn 1 $$2NaCl\left(aq\right)+H_{2}O\left(l\right)+CO_{2}\left(g\right)\to Na_{2}CO_{3}\left(s\right)+H_{2}\left(g\right)+Cl_{2}\left(g\right)$$

The developed variants of the CODA process, summarized in Figure 1, differ in absorber and crystallization configurations. The solubility curves of soda ash and its hydrates, both in the absence and presence of NaOH, were measured and modeled in the previous work.^8^ As the sodium carbonate system features three hydrates in the temperature range between 5 and 150 °C, two main crystallization pathways lead to Na_2_CO_3_ anhydrate: (1) a two-step route via Na_2_CO_3_·10H_2_O followed by Na_2_CO_3_·1H_2_O, and (2) direct monohydrate crystallization. Variant P1 uses a cross-flow packed absorber and provides decahydrate by evaporative cooling; P2 achieves supersaturation during CO_2_ absorption not requiring cooling but advanced absorber technology to manage potential crystal formation, e.g., a droplet absorber.^9,10^ In variant P3, monohydrate is directly crystallized postabsorption. P4 is similar to P1 but includes HCl production from electrolysis instead of H_2_ and Cl_2_. Therefore, this study provides a comprehensive analysis of the crystallization behavior of both hydrates. Process feasibility has been assessed based on energy consumption, equipment size, and heat integration, supported by Aspen Plus modeling.^6^

**Figure 1:**
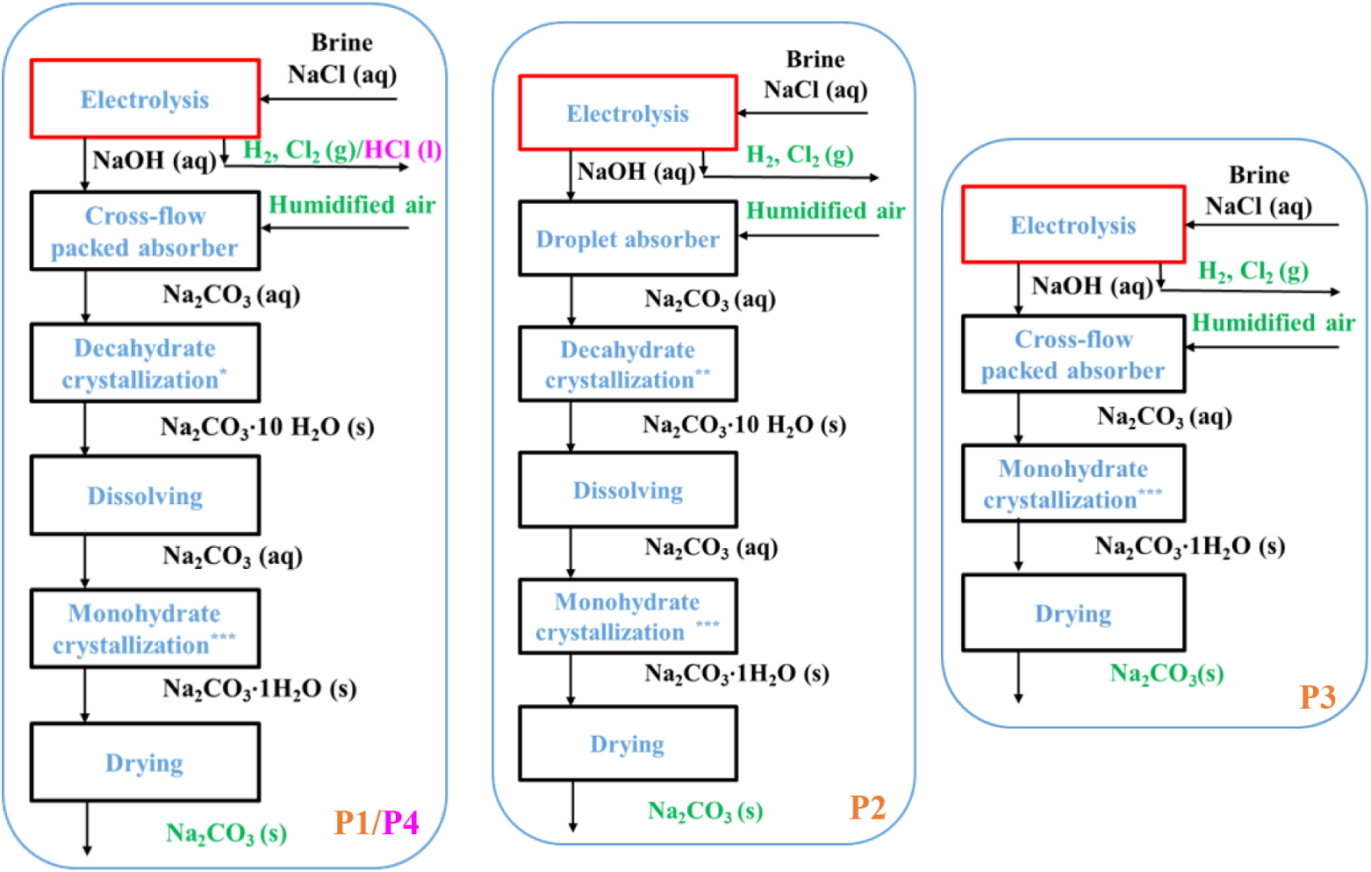
Schematic representation of the CODA process variants P1–4.^6^ Subprocesses with the highest energy consumption are highlighted in red boxes, while products, byproducts, and absorbed CO_2_ from air are indicated in green. (*Evaporative vacuum cooling crystallization, **Supersaturation generated in the absorber, ***Evaporative vacuum crystallization).

Direct crystallization of Na_2_CO_3_ anhydrate is generally avoided in industrial practice, primarily, as the crystals provided are significantly less compact than those produced from the monohydrate form. Additionally, the anhydrate’s stability region necessitates the use of costly and energy-intensive pressurized crystallizers. A mixed-solvent approach was proposed^11^ using >20 wt % ethylene glycol to raise the boiling point, enabling anhydrate crystallization at atmospheric pressure and producing dense soda ash (1050 kg/m^3^). NaOH also lowers the mono- to anhydrate transition temperature from 110.5 °C to 100 °C at 11.6 wt % NaOH.^12^ However, the CODA process avoids this route due to the high evaporation energy demands, and the anhydrate crystallization behavior under such conditions remains insufficiently studied.

Crystal growth kinetics is essential for controlling material properties, optimizing production, and ensuring product quality while minimizing waste and energy use. In recent studies Helfenritter and Kind applied a desupersaturation-based method using confocal micro-Raman spectroscopy to investigate Na_2_CO_3_·10H_2_O growth in a 150 μm thin film layer in Na_2_CO_3_–Na_2_SO_4_ systems.^13^ Shaikh et al.^14^ studied Na_2_CO_3_·1H_2_O growth during solution-mediated conversion of Na_2_CO_3_ to Na_2_CO_3_·1H_2_O, as it happens in the Monohydrate process. However, no prior work has examined these kinetics within the Na_2_CO_3_–NaOH–H_2_O system.

In this work, crystal growth kinetics of Na_2_CO_3_·10H_2_O and Na_2_CO_3_·1H_2_O are investigated within the Na_2_CO_3_–NaOH-H_2_O system in a 3 L-scale for the first time. Building on the solid–liquid equilibrium (SLE) models, metastable zone width measurements, and the used setup detailed in prior work,^8^ growth kinetics experiments were meticulously designed following a recently developed shortcut method.^15,16^ This shortcut method enables quantification of crystallization kinetics such as growth, nucleation, and dissolution rates without requiring the solution of full population balance equations and provides a fast and straightforward approach to determining kinetics, simplifying process design, and reducing experimental effort. This is accomplished by seeded batch crystallization experiments observing the crystal size evolution by employing mass balance modeling validated by offline crystal size distribution measurements. Growth kinetics of Na_2_CO_3_·10H_2_O were determined via cooling crystallization in the temperature range of 17–6 °C. Na_2_CO_3_·1H_2_O crystallization was examined using evaporative crystallization under vacuum conditions at 50 °C and a constant evaporation rate. In both cases, a 5 wt % NaOH concentration in the system was employed, identified as the optimal concentration for direct CO_2_ absorption in NaOH for the CODA process.^7^ The impact of NaOH on crystal growth and crystal morphology, as well as the influence of supersaturation degree on crystal quality, were also investigated.

## 2 Experimental Tools and Methods—Description and Evaluation

### 2.1 Materials and Reagents

NaOH (pellets), Na_2_CO_3_, and Na_2_CO_3_·10 H_2_O all with ≥99.0% purity, and HCl, 1 M, Titripur were purchased from Merck, Germany. NaCl and Na_2_CO_3_·1H_2_O with a purity of ≥99.0% from Sigma-Aldrich, Germany. Isopropanol and ethanol with ≥99.7% purity were supplied from VWR Chemicals, Germany. Deionized water from a Milli-Q system by Merck (Germany) was used in all experiments.

### 2.2 Applied Shortcut Method for the Quantification of the Crystal Growth

The concept of employing a shortcut method to measure the crystal growth rate under nonisothermal batch conditions was initially proposed by Yokota et al.,^17^ and this idea was further extended by Temmel et al.,^15,18^ to include the measurement of nucleation and dissolution rates. The method has been validated using in silico data with predefined kinetics, demonstrating its ability to recover specified parameters with limited input information.^16^ This streamlined approach is useful for estimating the kinetic parameters necessary for population balance models in crystallization processes. These models are crucial for designing, controlling, and optimizing crystallization processes by describing kinetic phenomena, such as growth, dissolution, and nucleation of particles. Traditionally, determining component-specific parameters for these models requires extensive experimentation for each substance system or experimental setup. The shortcut method simplifies this by analyzing the evolution of particle (or crystal) size distribution (PSD) during a few batch crystallization experiments. In this work, this method is applied exclusively to measure the growth rate. The procedure involves several key steps. First, a series of batch crystallization experiments are conducted under controlled conditions during which the PSD is observed at various stages. Changes in the PSD are then analyzed to estimate the kinetic parameters by examining the movement of the seed peaks resulting from growth. The observed experimental data, including temperature and component concentrations, are used to calculate the supersaturation level, which is essential for estimating parameters for kinetic models. This estimation is achieved by fitting the experimental data to predefined models that describe the growth kinetics. The principle of this method, as applied within this work, is illustrated in Figure 2.

**Figure 2:**
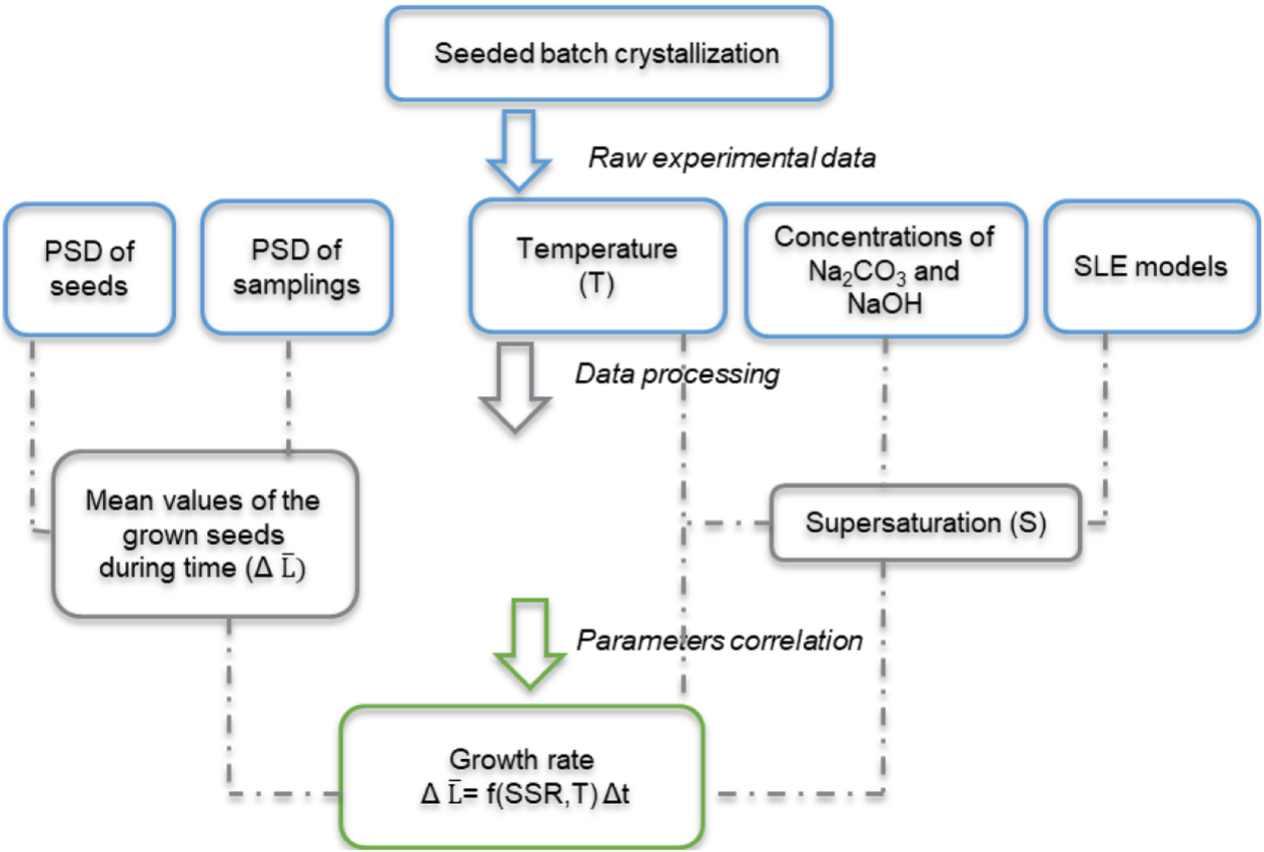
Principle of the shortcut method^18^ for measuring the crystal growth rate within this work.

The empirical equation, eq 2, with fitting parameters *k*_*g*_ and *g*, which is used mostly for industrial and engineering purposes^19^ was employed in this work as the growth rate model. This equation is derived from a diffusion-reaction model. It comprises only the two rate-determining general substeps of crystal growth: diffusive transport from the bulk phase to the crystal surface and subsequent lattice integration. In this equation *G* is the growth rate of length *L*, SSR is the supersaturation ratio, and *k*_*g*_ and *g* are fitting parameters. It should be noted that the exponent *g* is not clearly related anymore to the growth mechanism.^18^

1 $$G=\frac{dL}{dt}=k_{g}\left(T\right){\left(SSR\left(T\right)-1\right)}^{g}$$

The overall temperature dependency of eq 2 can be approximated with the well-known Arrhenius approach (eq 3).

2 $$k_{g}=k_{g,0}\;\exp\left(\frac{-E_{A,g}}{RT}\right)$$

Consequently, three fitting parameters, *k*_*g*,0_, *E*_*A*_,*_g_*, and *g*, were optimized by utilizing the experimental data. In optimizing the kinetic parameters, the objective function employs a least-squares method across the number of experiments (*n*_exp_) with five offline samples (*n*_samples_ = 5). In the case of using the crystal size obtained from mass balance, the number of samples increases to one sample each 30 s. The objective function is defined as follows:

3 $$OF_{G}=\overset{n_{Exp}}{\underset{j=1}{\sum }}\overset{n_{Sample}}{\underset{i=1}{\sum }}{\left({\mathrm{L̅}}_{sim,i,j}-{\mathrm{L̅}}_{\exp,i,j}\right)}^{2}$$

The values ${\mathrm{L̅}}_{sim}$ in these objectives can be calculated by

4 $${\mathrm{L̅}}_{sim}={\mathrm{L̅}}_{seed}+\int_{0}^{t}k_{g,0}\exp\left(\frac{-E_{A,g}}{RT}\right){\left(SSR-1\right)}^{g}dt$$

The parameters for the kinetics model were fitted only for experiments with NaOH and separately for the Na_2_CO_3_ deca- and monohydrate applying a simplex algorithm and a stochastic initial value generator.^18^ The initial parameters for the optimization are randomly generated by using functions in Matlab. These functions generate random integers and random floating-point numbers, respectively.

### 2.3 Experimental Setup

A 3 L-scale crystallization setup equipped for cooling and vacuum evaporative crystallization, that was already used in previous work,^8^ is applied to conduct the experiments for the crystal growth kinetics study. The flow diagram of this setup is shown in Figure 3 (left). This setup was designed to exclude cooling and vacuum evaporative crystallization experiments, based on the solubility curve of Na_2_CO_3_ hydrates shown in Figure 3 (right). The setup primarily consists of a 3 L-scale draft-tube baffled crystallizer, which is double-walled and connected to a thermostat to precisely control the process temperature. A vacuum pump (PC 3001 VARIO, Vacuubrand, Germany) provides the vacuum required for the evaporative crystallization; the entire setup is sealed gastight to maintain the desired vacuum pressure. A condenser, connected to a separate thermostat, is used to condense water vapor during vacuum operation. A balance is installed to continuously measure the mass of condensed water, enabling the precise monitoring of evaporation. For inline concentration measurements of NaOH and Na_2_CO_3_ in the liquid phase during both cooling and vacuum evaporative experiments, an FTIR probe (ReactIR 45m, Mettler Toledo) is employed. This device, equipped with a silver-halide optical fiber and a diamond optical window, enables attenuated total reflection Fourier transform mid-infrared (ATR-FT-MIR) spectroscopy in the wavenumber range of 1900 to 650 cm^–1^. Real-time data from all analytic probes and the balance are recorded using a custom Python code, allowing for synchronized monitoring of process parameters. An automated sampling system was employed in this setup to collect samples within the sampling bottle, which was connected to a vacuum pump to facilitate sample collection during vacuum evaporative crystallization experiments. A plug valve was installed on the tube connecting the sampling bottle and the vacuum pump. This valve allows the pressure to be increased to atmospheric levels after sampling, enabling the opening of the sample bottle and replacement with another bottle for subsequent samples. The collected sample was immediately filtered by using a vacuum pump filter to separate the liquid and solid phases of the suspension. The liquid was removed for analysis, while the solid was washed with an antisolvent to remove the mother liquor for further analysis.

**Figure 3:**
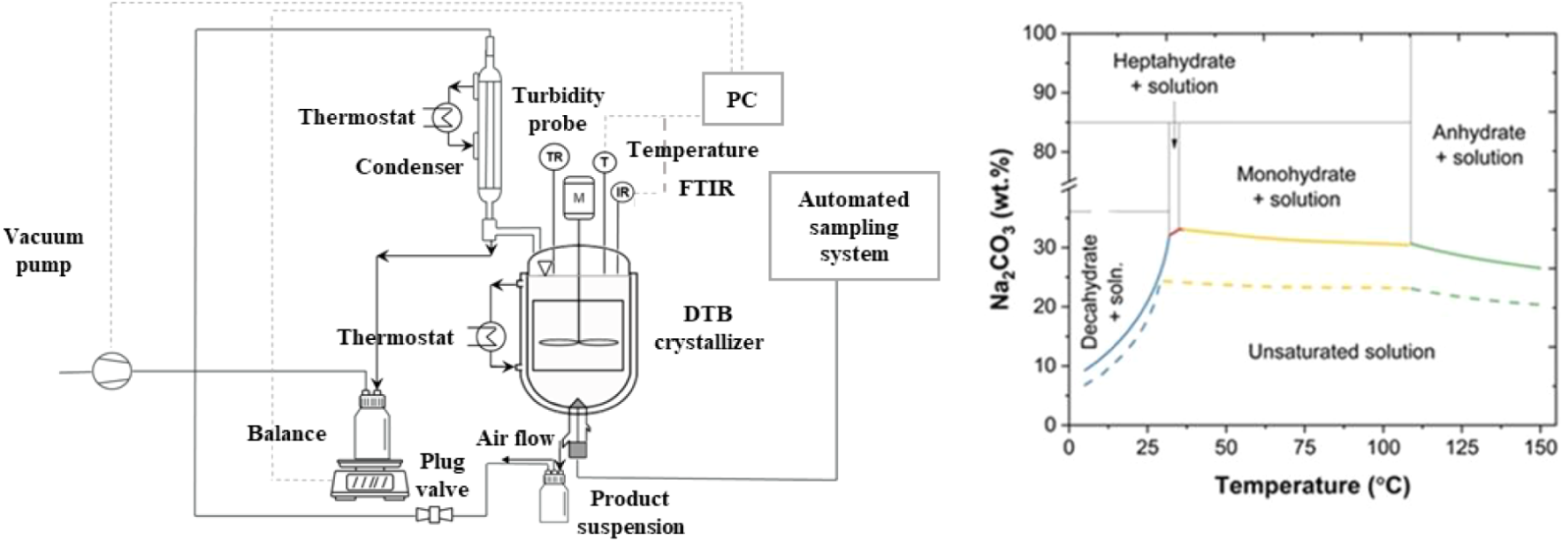
A flowchart of the crystallization setup used for cooling and evaporative crystallization with analytical probes (left). Solubility curve of soda ash hydrates, adapted from Gutierrez, M. F.; Lorenz, H.; Schulze, P. Carbon-Negative Production of Soda Ash: Process Development and Feasibility Evaluation. Chem. Eng. Ind. Chem. 2025, 64, 11474–11496, used under CC BY 4.0 (right).

### 2.4 Experimental Procedures for Growth Kinetics Determination

#### 2.4.1 Na_2_CO_3_·10H_2_O Growth Kinetics Measurements

Batch-seeded cooling crystallization experiments following a shortcut method were conducted for Na_2_CO_3_·10 H_2_O. The idea of this method is to provide several combinations of temperature and supersaturation during one experiment and therefore an efficient investigation of the desired kinetics over the considered range of process conditions. Approximately 3 kg of feed Na_2_CO_3_ solution was added to the crystallizer by applying vacuum pressure to draw the solution from the container into the reactor by using a tube. The solution was stirred at 600 rpm for about 30 min at 30 °C. Subsequently, it was cooled down at rates ranging from 1.19 to 4.17 K/h using a thermostat that operated with thermal G fluid, Julabo. Seeding was carried out within the metastable zone width (MSZW) at 17 °C, based on previously measured MSZW data,^8^ which were determined under identical conditions (same setup, concentration, cooling/evaporation rates, and agitation rates). Given the large MSZW (>10 K), preliminary trials indicated that subcooling promotes agglomeration, hindering reliable growth-rate determination. Therefore, we adopted a seeding strategy slightly before the saturation temperature; the resulting minor seed dissolution was accounted for in the modeling. The crystal images of subcooling tests with/without NaOH are provided in the SI (Figures S2–S3). While seeding, the saturated solutions were cooled down from temperatures around 17 to 6 °C, reaching a maximum suspension density of around 15–20% (depending on the presence of NaOH), which is close to industrial levels, reported in the range of 15–25%.^20^ The seed amount was around 6% of the expected final product yield. Details of the cooling crystallization experiments studied (only experiments with dominant crystal growth) are compiled in Table 1. As the seeds grew, periodic sampling was done depending on the cooling rate, ranging from every 30 min in Exp. 1 to every 2 h for the lowest cooling rate in Exp. 3. The obtained suspension samples were immediately filtered via a vacuum filter to separate the solid and liquid phases for subsequent analysis. Isopropanol was used to wash the filtered decahydrate. Ethanol was also tested for washing; however, it caused the crystals to break. Exps. 1 and 2 with different cooling rates were conducted to provide a robust set of estimated parameters; Exp. 3 was designed to investigate the effect of cooling rate on crystal growth behavior, based on the findings from Exps. 1 and 2. It should be noted that conducting each experiment at different temperature ranges instead of different cooling rates would result in varying sets of NaOH and temperature points, complicating the FTIR concentration calibration. For the estimation of kinetic parameters, the results of two experimental runs were used for each hydrate phase under NaOH-containing conditions (reported in Table 4). Exps. 2 and 3 were identical except for the cooling rate, confirming reproducibility through consistent SSR values (Figure S10). Additionally, initial test experiments showed reproducible concentration data for both hydrates, although PSD measurements were not performed microscopically due to workload constraints. Only one experiment, Exp. 4, was conducted without NaOH to demonstrate the effect of NaOH on crystal growth rate and crystal morphology. Hence, kinetic parameters were not estimated for this experiment. The stirring rate in all experiments was 600 rpm. Supersaturations were calculated based on the SLE models derived in the previous work^8^ for each hydrate.

**Table 1:** Details of the Cooling Crystallization Experiments Including Saturation Concentration of Na_2_CO_3_ $\left(C_{{\mathrm{Na}}_{2}CO_{3}}\right)$ , Concentration of NaOH (*C*_NaOH_), and the Corresponding Saturation Temperatures (*T*_eq_), Solution Amount, Seed Size Fraction and Amount, Temperature at Seeding Point (*T*_seed_), Cooling Rate (CR), and Subcooling Degree at Seeding Point (*T*_eq_ – *T*_seed_) for the Crystal Growth Rate Measurements of Na_2_CO_3_·10H_2_O via Cooling Crystallization

Exp.	$C_{{\mathrm{Na}}_{2}CO_{3}}$ (wt %)	*C*_NaOH_ (wt %)	*T*_eq_ (°C)	Solution amount (g)	Seeds size fraction (μm)	Seed amount (g)	*T*_seed_ (°C)	CR (K/h)	*T*_eq_ – *T*_seed_ (°C)
1	11.48	4.87	16.69	3000.8	250–300	31	16.91	4.14	–0.22
2	11.44	4.81	16.58	3088.4	250–300	30.82	16.62	2.58	–0.04
3	11.51	4.96	16.80	2809.8	250–300	31.08	16.89	1.19	–0.09
4	15.36	0	16.24	3024.8	250–300	34.73	16.48	4.17	–0.24

#### 2.4.2 Na_2_CO_3_·1H_2_O Growth Kinetics Measurements

As shown in Figure 3 (right), the solubility of Na_2_CO_3_·1H_2_O is almost invariant with temperature in its existence region (∼35–110 °C), and the hydrate maintains stability below the boiling point of its aqueous solution under atmospheric pressure.^12^ Consequently, vacuum evaporative crystallization at a constant temperature and evaporation rate was utilized to assess the crystal growth kinetics. As above, about 3 kg of the Na_2_CO_3_ feedstock solution was introduced into the crystallizer. The target constant temperature for evaporative crystallization, 50 °C, was sustained via a controlled-temperature jacket. This temperature was chosen because lower temperatures require less energy, which is advantageous for industrial applications. However, the temperature was not set too low to avoid the formation of an intermediate hydrate phase and to avoid slow growth kinetics. To ascertain the corresponding saturation vapor pressure of binary or ternary aqueous mixtures of NaOH, Na_2_CO_3_ and H_2_O at the specified temperature, the pressure was initially set to the saturation vapor pressure of pure water at the given temperature. Subsequently, the pressure was incrementally reduced until the formation of small evaporation bubbles within the crystallizer was observed. This pressure was then identified as the saturation vapor pressure of the solution, and the evaporation rate was modulated by adjusting the external temperature in the reactor’s controlled-temperature jacket using a thermostat. At this juncture, the thermostat’s control mechanism was switched from crystallizer to jacket temperature to ensure a constant evaporation rate. The evaporation rate was determined by continuously monitoring the data from the condensate water balance. Given the fixed applied vacuum pressure, the temperature within the reactor escalates due to alterations in saturation vapor pressure induced by concentration changes, resulting in a temperature increase within the crystallizer. To maintain a constant internal temperature, the applied pressure was manually decreased periodically. The obtained average temperature throughout the experiments, along with the corresponding standard deviation, is documented in Table 2. According to prior research,^8^ at 50 °C across varying evaporation rates the MSZW was found to be independent of the evaporation rate at approximately 1.10 supersaturation ratio in the presence of 5 wt % NaOH and at about 1.08 without NaOH. As depicted in Table 2, seeds were introduced within the measured MSZW, and sampling occurred approximately every 30 min as the seeds grew. For seeding, the vacuum pressure was halted and seeds were introduced into the reactor. The setup was equipped with an automated sampling system, integrated with the vacuum system, facilitating sampling under vacuum pressures. An evaporation rate of about 3 g/min was employed to mitigate the excessive bubble formation that could interfere with analytical probes. A smooth evaporation process also prevents the deposition of dried carbonate on the walls due to large bubbles contacting the surfaces. To achieve varying supersaturation levels, different seed quantities were employed in Exps. 5 and 6; the seed amount was around 8% of the expected final product in Exp. 5. However, the experiment without NaOH (Exp. 7) was conducted with a single seeding quantity to elucidate the effect of NaOH on the crystal growth rate and crystal morphology. The experiments reported in Table 2 showed dominant crystal growth, i.e., no nucleation or agglomeration caused by elevated supersaturation. Ethanol was used for washing the filtered monohydrate samples since isopropanol caused the monohydrate crystals to stick together. The stirrer speed used in all experiments was 770 rpm, which ensured full suspension of the slurry without affecting the PSD.

**Table 2:** Details of the Vacuum Evaporative Crystallization Experiments Including Saturation Concentration of Na_2_CO_3_ $\left(C_{{\mathrm{Na}}_{2}CO_{3}}\right)$ , NaOH Concentration (*C*_NaOH_), Solution Amount, Seed Size Fraction and Amount, Supersaturation at Seeding Point (*S*_seeding_), Evaporation Rate (ER), Average Temperature (*T*_avg_) and the Standard Deviation of the Average Temperature (SD of *T*_avg_) for the Crystal Growth Rate Measurements of Na_2_CO_3_·1H_2_O via Vacuum Evaporative Crystallization

Exp	$C_{{\mathrm{Na}}_{2}CO_{3}}$ (wt %)	*C*_NaOH_ (wt %)	Solution amount (g)	Seeds size fraction (μm)	Seed amount (g)	*S*_seeding_ (min)	ER (g/min)	*T*_avg_ (°C)	SD of *T*_avg_
5	24.52	5	3086.8	212–250	16.54	1.052	2.88	49.84	0.17
6	24.61	5	3215.4	212–250	10.27	1.055	3	49.79	0.27
7	31.52	0	3206.7	212–250	16.99	1.07	2.89	49.73	0.16

#### 2.4.3 Preparation of Seed Crystals

Na_2_CO_3_·10H_2_O and Na_2_CO_3_·1H_2_O seed crystals were derived from a commercially acquired material. They were selected from a specific particle size fraction obtained through sieving, without any milling process, with the fraction sizes detailed in Tables 1 and 2. Given that Na_2_CO_3_·10H_2_O is thermodynamically unstable under ambient (dry) conditions and tends to lose water during the sieving process, it was stored in a desiccator containing saturated NaCl solution to facilitate the reabsorption of water postsieving. More detailed information can be found in Figure S4.1, Figure S4.2. In contrast, Na_2_CO_3_·1H_2_O remains stable at room temperature, and thus, the seeds required no additional treatment postsieving. Images of the seed crystals, captured by a digital microscope, are depicted in Figure S4.2 in the SI.

### 2.5 Analytical Procedures

#### 2.5.1 Analysis of Liquid Phases: Titration and FTIR Spectroscopy

The liquid phases in the Na_2_CO_3_–NaOH–H_2_O system were analyzed by using titration as an offline method alongside an inline method employing an FTIR probe. The titration method using Eco-Titrator Acid/Base (Metrohm) is described in previous work.^8^ Offline concentration measurements were used to calibrate the FTIR inline analyses. Calibration details and the off- vs inline agreement plot are provided in the SI (Section S5, Figures S5.1 and S5.2).

#### 2.5.2 Analysis of Solid Phase: Hydration Levels, Morphology, and Crystal Size Evolution

The titration method was employed to determine the hydration levels of the seeds, as well as those of the decahydrate and monohydrate products as explained in the previous work.^8^ To analyze the morphology of the crystals and to obtain the PSD of the solid samples, a VHX-2000E digital microscope (Keyence, Japan) was utilized. Tanga and Libowitzky^21,22^ revealed that Na_2_CO_3_·10H_2_O has a monoclinic crystal structure. Figure S6, SI, illustrates single crystals of Na_2_CO_3_·10H_2_O grown in the presence of NaOH, obtained by slow evaporation at room temperature. Two crystal habits were observed: rhombic and hexagonal prisms, both grown as platelets. The term crystal habit refers to the typical external appearance of a crystal, which depends on the relative development of the crystal faces. In contrast, crystal morphology describes the complete geometrical form arising from the crystal’s symmetry and face arrangement. Figure 4 shows the schematic representations of the crystal morphologies obtained in this work for both hydrates, along with the corresponding length designations: *L*_1_ (minimum length) and *L*_2_ (maximum length) of the hexagonal faces, and *L*_3_ (prism height). The length H, defined for the monohydrate, is used solely to calculate the volume of the irregular hexagonal prism. In all experiments conducted in this study, only hexagonal prisms were observed (Figure 4 (left)), except for one preliminary experiment without NaOH that is shown in the SI, Figure S3b, which was excluded in this work due to nucleation and agglomeration.

**Figure 4:**
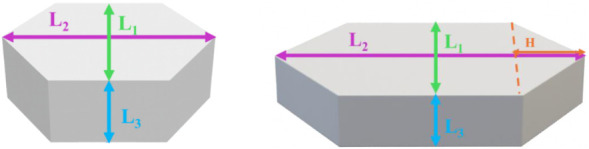
Crystal morphologies and corresponding defined lengths of the obtained regular hexagonal prism for Na_2_CO_3_·10H_2_O within cooling crystallization (left), and the irregular hexagonal prism obtained for Na_2_CO_3_·1H_2_O within vacuum evaporative crystallization in the system of Na_2_CO_3_–NaOH–H_2_O (right).

As ref. 23 reveals, Na_2_CO_3_·1H_2_O has an orthorhombic hemimorphic space group. The only habit obtained for this hydrate here was irregular hexagonal prisms, as shown in Figure 4 (right), which are commonly referred to as coffin-shaped crystals in the literature^24,25^ . The single crystal grown by slow evaporation at 50 °C in the presence of NaOH had the same habit.

Particle size distribution (PSD) was determined from digital micrographs acquired with a Keyence VHX-2000E microscope using a 50× lens. The system was calibrated with the OP-87426/87427 reference scale, and multiple overlapping images were automatically stitched to obtain wide-field, high-resolution views containing several hundred crystals each. The software’s automated area-measurement algorithm, based on brightness contrast, provided particle metrics such as area and characteristic diameters. The maximum length was defined as the greatest Euclidean distance within the particle perimeter and the minimum length as the shortest distance between parallel tangents. Objects smaller than 50 μm, touching particles, and those at the image borders were excluded. For each sample, about five stitched images (≈2000 particles) were analyzed to ensure statistical robustness. It should be noted that the digital microscope was not equipped for 3D analysis.

To determine the PSD of the samples, the raw length measurement data obtained from the digital microscope were analyzed using a custom MATLAB script. This analysis yielded the frequency distribution based on particle count (*q*_0_). The progression of crystal growth was monitored by calculating the mean crystal size from the normalized Gaussian distribution of the seed crystals and by observing the growing seeds, eliminating dust or fine particles, as well as agglomerated large particles. The PSD was derived from the maximum particle length for decahydrate, whereas for the monohydrate samples it was based on the minimum particle length. This distinction is crucial in cases of star-shaped agglomeration for monohydrate, where the minimum length remains representative of length *L*_1_, but the maximum length no longer accurately represents length *L*_2_ in Figure 4 (right).

It is important to note that when analyzing the PSD of Na_2_CO_3_·10H_2_O crystals, they tend to fracture easily when completely dry. Thus, the samples were immersed in isopropanol to facilitate easier distribution under the microscope lens. However, these samples are transparent inside solution and the automated area measurement function, which relies on particle brightness, cannot effectively distinguish this hydrate. Therefore, the samples were allowed to dry slightly to acquire a whitish hue, rather than remaining transparent, but not to the extent of becoming brittle. Therefore, they were exposed to air for only a few minutes prior to commencing microscopic analysis. Occasionally also a PVM (Particle Vision and Measurement) probe (Mettler-Toledo, Germany) was applied to qualitatively follow and evaluate the particle size and shape evolution inline.

## 3 Results and Discussion

### 3.1 Elevated Crystal Size Evolution Based on the Mass Balance Modeling

The evolution of seed size was modeled by using a mass balance approach based on inline Na_2_CO_3_ concentration measurements. This modeling served two main purposes: (i) to confirm that the experiments were conducted under non-nucleation conditions, and (ii) to evaluate the mean crystal size obtained from PSD measurements using optical microscopy. This comparison was intended to determine whether the mean crystal size from microscopy or from mass balance modeling should be used for the estimation of kinetic parameters. To ensure that growth was predominant across all experiments, a Focused Beam Reflectance Measurement (FBRM) probe was tested. However, due to the substantial size of the crystals in both decahydrate and monohydrate forms, the data obtained were neither representative nor reliable. Consequently, the evolution of seed size was modeled based on a mass balance. In this idealized model, it is assumed that all seeds grow at a uniform rate, secondary nucleation is absent, and all seeds possess identical habits and sizes, following a normal (Gaussian) size distribution. The seeds’ number was estimated from seed mass and habit; crystal size was back-calculated every 30 s from the Na_2_CO_3_ mass balance using literature densities, 1.46 and 2.25 g/cm^3^, for decahydrate and monohydrate, respectively.^26^ In the case of monohydrate, the measured condensed water with a balance was used alongside the inline concentration measurements. Two crystal morphologies were assumed for the seeds and the resulting product crystals of decahydrate: one spherical and the other a regular hexagonal prism. For the monohydrate, only irregular hexagonal prisms are assumed for the seeds and the grown crystals. The proportion between the dimensions of the prism was calculated based on the defined characteristic length (*L*_2_ decahydate and *L*_1_ for monohydrate) for each experimental set, depending on the presence or absence of NaOH. Hence, the volume of the crystals can be calculated only based on one length. Table 3 illustrates the proportions between the lengths, derived from only the final samples only. For decahydrate, the presence of NaOH does not affect the crystal habit but influences that of monohydrate, causing the crystals to elongate the dimension *L*_2_. The model also accounts for the effect of suspension loss due to sampling in both the liquid and solid phases. The sample sizes used for the model are not based on offline measurements with digital microscope but on crystal size 30 s prior to sampling obtained with the mass balance. Thus, the number of crystals in the sampled suspension is calculated based on its size and subtracted from the total number of seeds that were growing. Figure 5 presents the modeling results for Exp. 1 for both assumed morphologies, along with the mean particle sizes obtained from digital microscopy. The red bars represent the standard deviations (σ), width of the fitted Gaussian size distributions. The selected crystal morphology determines the number of seeds, as the seeds of Na_2_CO_3_·10H_2_O depicted in the SI, Figure S4.2a, are neither purely spherical nor regular hexagonal prisms; both morphologies are considered. As shown in Figure 5, both the spherical and prismatic models fall within the experimental standard deviation range; however, the mean particle sizes show slightly better agreement with the spherical model, which is retained for subsequent analyses due to its simpler geometric assumptions, as illustrated in Figures 7 and 9. In Figure 5, the reduction in size in the beginning is due to the small “minus” subcooling degree before seeding. The crystal sizes obtained from mass balance modeling had the most deviation from sizes measured via microscope within Exp. 2. To ensure no significant concentration loss due to the nucleation or the growing crystalline fines within the seeds, the model calculated the reduction in size assuming a total 10% concentration loss, due to continuous secondary nucleation from the experiment’s onset. In this scenario, the SSE of errors was a minimum for the last two samples. This minor concentration loss validates all measurement methods and model assumptions, confirming that growth predominated over nucleation, breakage, and attrition, even at higher suspension densities. Figure S7 in the SI illustrates this model for Exp. 2, with results compared to offline crystal size measurements. As shown, the first two samples are slightly larger than the model predicts, suggesting that the error may be related to the size measurements of decahydrate crystals conducted in the antisolvent. A slight shadow of crystals was observed in the solution, which was considered as the crystal size. This was mainly because decahydrate crystals reflect light at certain angles, causing glare or bright spots that obscure the true outline. Additionally, a small fraction of particles was not perfectly oriented on their hexagonal faces during imaging. For Na_2_CO_3_·10H_2_O this misorientation could lead to an up to ∼7% overestimation of *L*_1_: when particles are not perfectly oriented on their hexagonal faces, the microscope measures √(*L*_1_^2^ + *L*_2_^2^) as the maximum diameter instead of *L*_2_. To mitigate all the mentioned errors, the modeling employed a spherical-equivalent morphology, using only the seed sizes obtained from microscopy. The sizes derived from the modeling were then used to correlate the kinetic parameters. For the monohydrate, there is strong agreement between the crystal sizes measured offline and those back-calculated from solution concentrations based on mass balance, as shown later in Figures 11 and 12. It should be noted that for Na_2_CO_3_·H_2_O the misorientation during imaging could lead up measuring *L*_3_ instead of *L*_1_ as the minimum length; however it does not affect the determination of the mean particle size based on *L*_3_ rather than *L*_1_, since *L*_1_/*L*_3_ ≈ 2, and the standard deviations of the distributions did not exceed 200 μm. This deviation would be too large to fall within the Gaussian distribution and, therefore, does not compromise the analysis.

**Table 3:** Proportions between the Lengths of the Assumed Crystal Morphologies Based on the Dimensions Presented in Figure 4 (right and left), for Both Na_2_CO_3_ Decahydrate and Monohydrate

	Decahydrate	Monohydrate			
Exp.	*L*_2_/*L*_3_	*L*_2_/*L*_1_	*L*_3_/*H*	*L*_1_/*H*	*L*_3_/*L*_2_
1–3	2.63	-	-	-	
5, 6	-	5.035	0.979	1.77	0.1098
7	-	2.579	0.604	1.666	0.145

**Figure 5:**
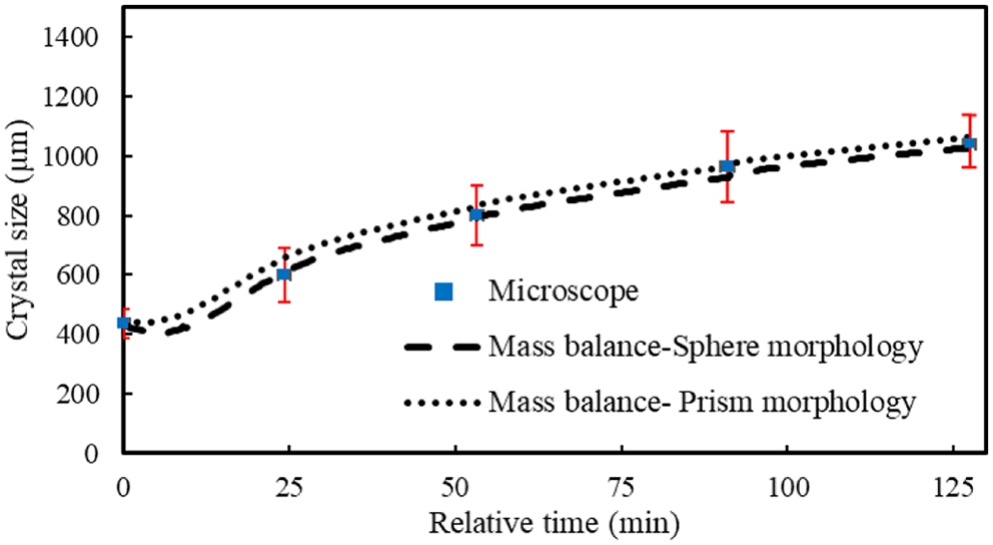
Crystal size evolution of Na_2_CO_3_·10H_2_O derived from mass balance calculations for Exp. 1, assuming two different morphologies: spherical and regular hexagonal prism. Red bars indicate the standard deviations (σ), which represent the width of the Gaussian distributions for each sample.

### 3.2 Growth Rate and Quality of Na_2_CO_3_·10H_2_O Crystals

Figure 6 exemplarily illustrates the progress of the cooling crystallization process conducted in Exp. 2, detailing the inline-measured temperature, Na_2_CO_3_ and NaOH concentrations, the calculated supersaturation ratio, and the crystal size development obtained from microscopic pictures. As derived, upon initiating cooling and introducing seeds into the reactor, the concentration of Na_2_CO_3_ begins to decline due to the crystallization of Na_2_CO_3_·10H_2_O. This reduction in Na_2_CO_3_ and water within the liquid phase results in an increase in the NaOH concentration. The supersaturation ratio is defined as the ratio of the current concentration to the saturation concentration at a given temperature and NaOH concentration. As illustrated in Figure 6, the supersaturation ratio increases from 0.99 at the seeding point to approximately 1.15. The obtained data on the temperature, supersaturation ratio, and crystal sizes were used to correlate the kinetic parameters.

**Figure 6:**
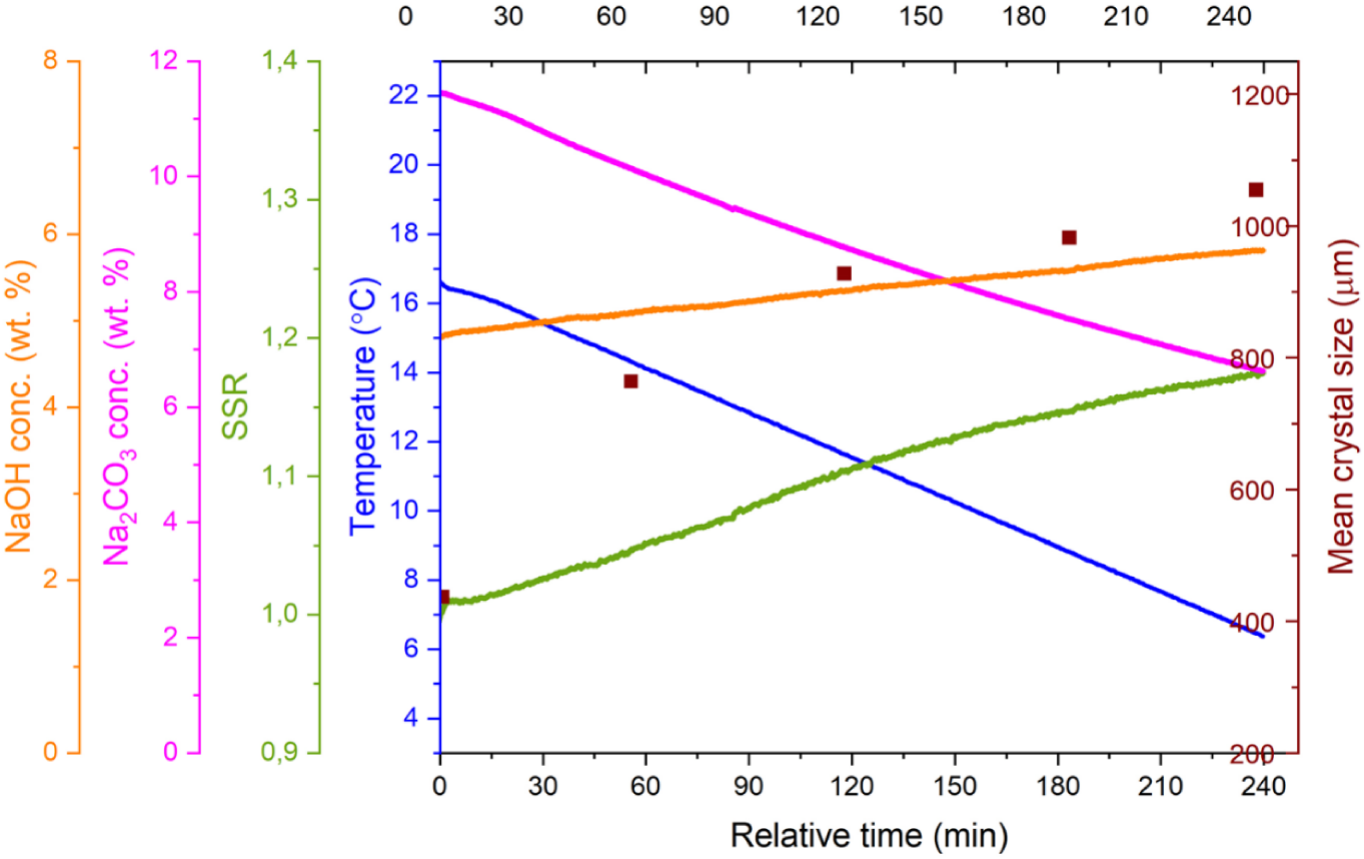
Experimental data from Exp. 2 (*t* = 0 seeding point) including the measured temperature (uncertainty of ±0.05 °C), in-line concentration of Na_2_CO_3_ and NaOH obtained by FTIR, the calculated supersaturation ratio, SSR, (relative uncertainty of 1%), and offline crystal size measurements conducted using a digital microscope based on maximum length.

Figure 7 presents the crystal growth rate of Na_2_CO_3_·10H_2_O from Exp. 3, as a function of the supersaturation ratio. The fluctuations correspond to the concentration measurements obtained by FTIR, from which the crystal sizes were derived. As shown, the growth rate initially rises with increasing supersaturation ratio, then gradually declines, and eventually stabilizes, even as the supersaturation ratio continues to increase up to approximately 1.15. According to classical crystallization theory, the growth rate typically increases with supersaturation, as also described in eq 2. A similar trend was observed across all experiments (Exps. 1–4). To ensure the reliability of this trend, a comprehensive validation of all relevant parameters was carried out. Therefore, a precise solubility model, developed in a previous study,^8^ was applied. The model covers the full range of temperatures and NaOH concentrations used within this work, ensuring accurate calculation of supersaturation ratios. Temperature measurements were carefully calibrated by using independent probes to ensure accuracy across all experiments. Na_2_CO_3_ concentrations determined via FTIR were further confirmed by mass balance calculations, using the NaOH concentrations from FTIR along with the measured sampling amounts of both liquid and solid phases. These results were fully consistent with the original FTIR data. Additionally, the titration results confirmed the FTIR-based concentrations, supporting the reliability of the concentration data. With all major data sets independently validated, a detailed analysis of crystal morphology was conducted. Figure 8 presents a comparison between a commercial Na_2_CO_3_·10H_2_O sample (Merck) and the crystals produced in this study, providing insight into the differences in the crystal appearance. The crystals produced in this work exhibit a partially hollow appearance, which initially raised the hypothesis of liquid inclusion, indicating that residual mother liquor might be trapped within the crystal. For verification, the solid phase was carefully analyzed using titration after drying and dissolving the crystals, and no NaOH was detected. This result excludes the presence of trapped mother liquor and strongly suggests that the internal appearance is a consequence of surface-related growth phenomena, particularly under conditions of high supersaturation. It should be noted that the same crystal quality was observed using an inline PVM probe; therefore, the partially hollow appearance cannot be attributed to dehydration during sampling or postprocess imaging.

**Figure 7:**
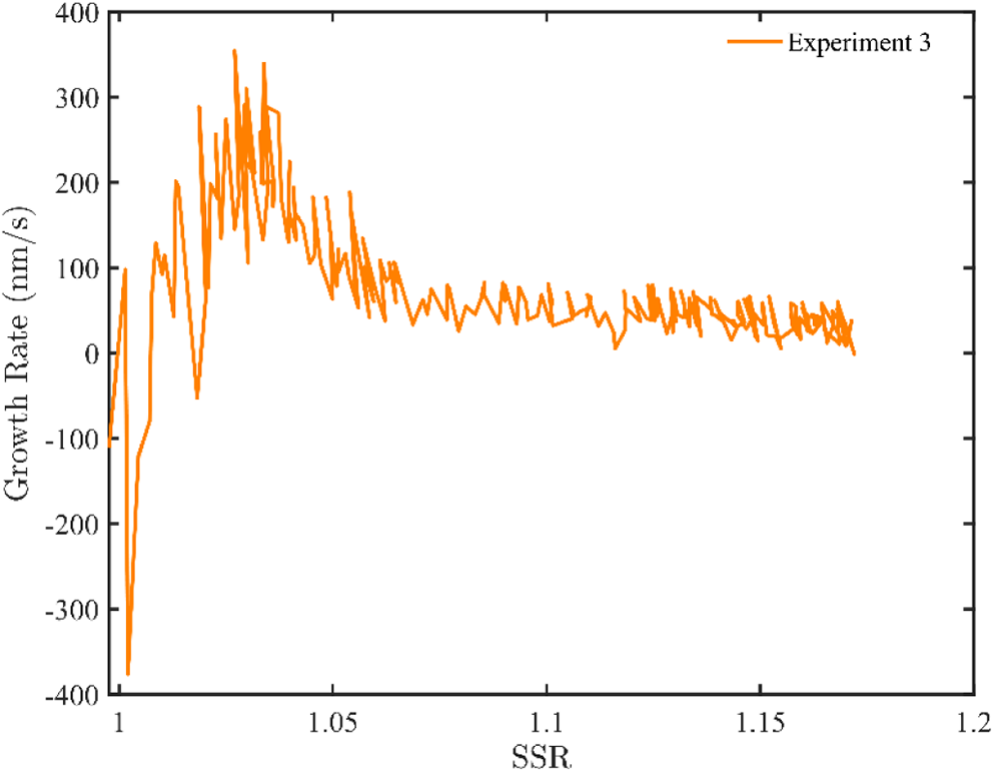
Crystal growth rate based on maximum length of Na_2_CO_3_·10H_2_O as a function of supersaturation ratio, SSR, (relative uncertainty of 1%), (Exp. 3).

**Figure 8:**
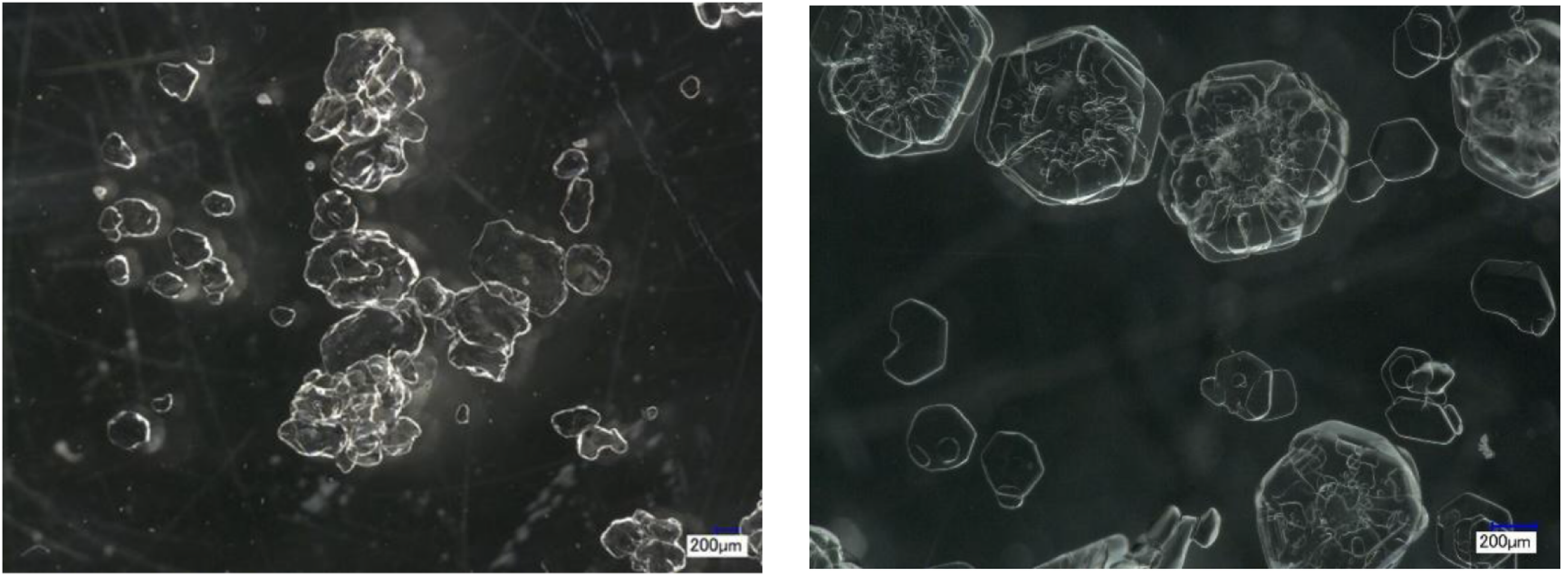
Digital microscope images of Na_2_CO_3_·10H_2_O: commercial from Merck, Germany (left), obtained in the presence of NaOH in this work (right).

Interestingly, in a similar respect, Pantaraks and Flood^27^ experimentally demonstrated in a 2-L batch crystallizer and later discussed^28^ that, beyond a certain point, further increases in supersaturation fail to enhance the crystal growth rate of sucrose, even in the absence of secondary nucleation. This phenomenon occurs beyond a critical supersaturation, where the crystal surface begins to degrade due to the imperfect incorporation of growth clusters, resulting in surface roughening. The effect becomes more pronounced at high growth rates and with prolonged residence times, as commonly encountered in batch systems. The transition referred to as the macroscopic roughening transition has been identified at relatively low supersaturation levels; for sucrose, this occurs between supersaturation ratios of 1.025 and 1.036.^27^ At supersaturation levels above 1.036, growth rates approach zero order, which is attributed to the accumulation of surface defects from previous high-growth conditions. Since our experiments were conducted under non-nucleating, impurity-free conditions, the observed decline in growth rate can be most explained by this surface-growth rate feedback mechanism.

The deviation in crystal growth behavior from classical expectations may be attributed to several hypothetical kinetic and structural factors. One possible explanation for the internal features observed, such as sector boundaries, hollow cores, and regions of inhomogeneous density, is face-dependent growth kinetics. In this process, different crystallographic faces grow at unequal rates due to anisotropic attachment kinetics, particularly under conditions of increasing supersaturation.^29^ At high supersaturation, some faces may become saturated or kinetically inhibited, resulting in an anisotropic growth behavior. This leads to sectoral zoning, where distinct growth sectors form with variations in composition or density, giving rise to visible sector boundaries. These boundaries mark transitions between regions governed by different crystallographic orientations and are manifested under microscopy as patterned internal textures. Importantly, such zoning does not require impurity incorporation and can occur even in chemically pure systems, highlighting its origin in intrinsic growth dynamics.^29^ In this work in situ PVM imaging and postsampling microscopy revealed irregular, nonuniform crystal surfaces and growth fronts, consistent with locally anisotropic face growth. These observations qualitatively support the proposed hypothesis of sectoral or face-dependent growth behavior, although they do not provide sufficient resolution to prove it. Another important mechanism influencing the growth behavior and internal structuring of Na_2_CO_3_·10H_2_O crystals is related to the intrinsic structural complexity of this highly hydrated salt. The crystal lattice incorporates a large number of water molecules, requiring precise spatial reorganization of the hydration layers during growth. At elevated supersaturation or under fluctuating temperature conditions, the rate of ion incorporation can exceed the rate at which the water network or hydration layers can rearrange within the growing lattice. This mismatch introduces a kinetic bottleneck, potentially resulting in misalignment, local structural inhomogeneities, or internal defects that become embedded as growth proceeds.^13,29^

Together, these factors offer a plausible explanation for the complex internal morphologies and, consequently, the observed deviation in crystal growth behavior from classical expectations even in the absence of additives such as NaOH. It is important to note that these possible interpretations remain hypothetical in the context of the present study, as direct evidence for the underlying mechanisms has not been obtained and is far beyond the objective of this study.

The development of the supersaturation ratio and crystal size of Na_2_CO_3_·10H_2_O in Exps. 1–4 is presented in Figure 9 (left). The observed trend aligns with expectations regarding experiments conducted at different cooling rates: the fastest cooling rate results in the highest crystal growth rate. The small steps observed in the crystal size evolution curves in Figure 9a are attributed to the sampling process, as each sample removed accounted for up to 1.5% of the reactor’s total mass. Figure 9 (right) shows concentration change rates for the related cooling experiments. As shown, all experiments exhibit a distinct turning point corresponding to the maximum growth rate. Notably, even the experiment conducted at the lowest cooling rate (Exp. 3) exhibits this behavior. This particular experiment was designed to investigate whether allowing more time for molecular rearrangement specifically the reorganization of water molecules within the crystal lattice would improve the growth rate. The hypothesis was that imperfect growth might be due to the ion incorporation rate exceeding the rate at which hydration layers or the water network can reorganize within the growing crystal. As mentioned above, the supersaturation value at which this decline begins is named critical supersaturation.^28^ For Exps. 1, 2, and 3, the critical supersaturation ratio values were found to be all around 1.03 regardless of the applied cooling rates, representing the highest supersaturation levels up to which the growth rate continues to increase with increasing supersaturation. Beyond this point, further increases in supersaturation do not enhance growth, and the growth rate becomes zero order. A comparison between Exps. 1 and 4, carried out under identical conditions except the absence of NaOH in Exp. 4, shows (Figure 9 (right)) that the latter also exhibits a turning point in the Na_2_CO_3_ concentration change rate, indicating that the decline in growth rate observed in Exps. 1–3 is not attributable to the presence of NaOH. Additional information showing the crystal mass produced over time for all experiments is provided in the SI with Figure S8.

**Figure 9:**
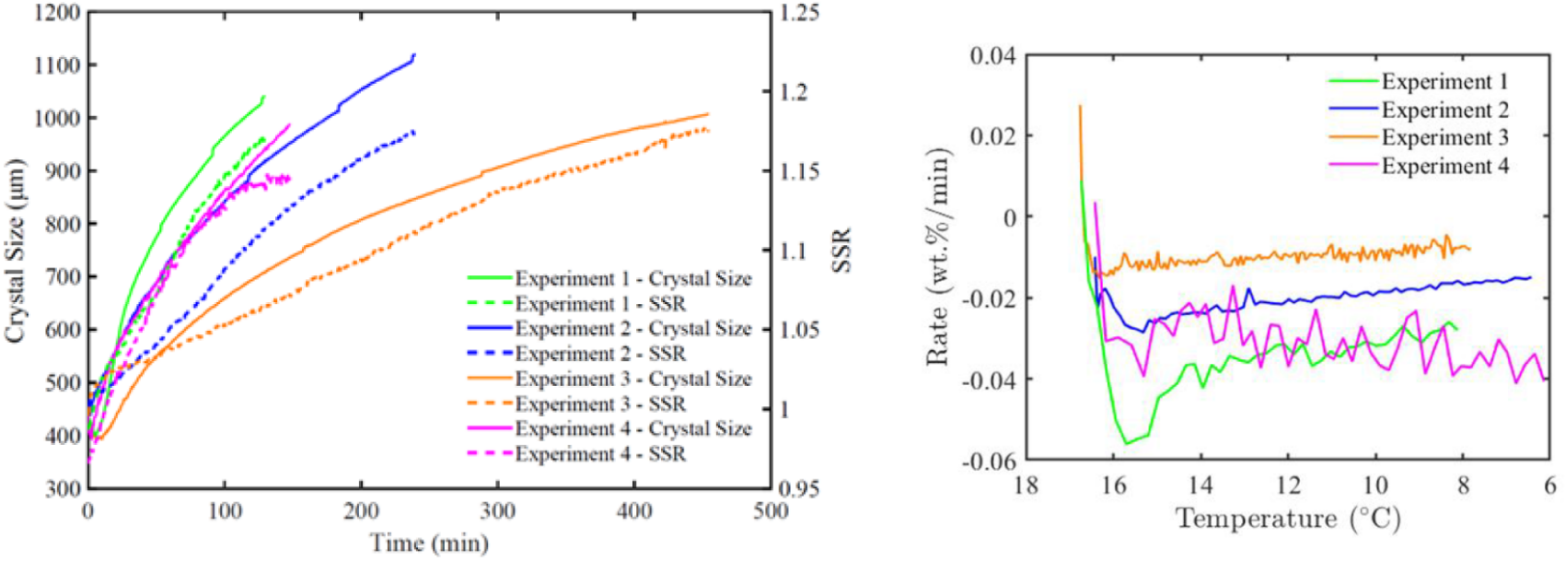
Progress of crystal size development of Na_2_CO_3_·10H_2_O as calculated from the mass balance and the corresponding supersaturation ratio, SSR, (relative uncertainty of 1%), (*t* = 0 seeding point) for Exps. 1–4 (left), Rate of Na_2_CO_3_ concentration change vs temperature for Exps. 1–4 (right).

### 3.3 Growth Rate and Quality of Na_2_CO_3_·1H_2_O Crystals

Figure 10 illustrates the results of Exp. 5, which is representative of the vacuum evaporative crystallization experiments conducted in the presence of NaOH. Seeds were added at time zero (*t* = 0), after the evaporation process had already commenced. At the point of seeding, the supersaturation ratio reached approximately 1.05, placing the system within the MSZW for sodium carbonate monohydrate. As evaporation progressed before seeding, the Na_2_CO_3_ concentration steadily increased. Following seeding, this upward trend continued briefly with a slightly decreased rate, peaking around 20 min, before a noticeable decline occurred. To verify whether crystallization was already taking place during this phase, Figure 11 presents a theoretical concentration curve assuming evaporation without any crystallization. This theoretical profile was calculated from the mass of condensed water collected, offering a reliable benchmark for comparison. Prior to seeding, the measured and calculated concentrations overlap, validating the accuracy of the FTIR-based in-line concentration measurements. After seeding, a slight deviation to lower concentrations appears, implying limited crystallization occurring initially, likely due to a delay in seed surface activation. This interpretation is further supported by a separate experiment using ten times the seed mass, which showed the same initial lag, suggesting that the delay is not due to insufficient seeding or surface area. After reaching a peak Na_2_CO_3_ concentration, a sharp drop is observed. This marks the moment when the crystallization rate surpasses the rate of evaporation, leading to a rapid decrease in supersaturation. The observed peak concentration corresponds closely to the previously reported MSZW thresholds: around 25.7 wt % at 50 °C with 5.5 wt % NaOH, and 34.1 wt % in the absence of NaOH. Following the trend of concentration change, the supersaturation value also increases after seeding and then due to the activated seeds, leads to rapid crystallization. Once crystallization is initiated, the supersaturation ratio drops quickly until it stabilizes at a nearly constant level where crystallization and evaporation are balanced. A slightly elevated final supersaturation level is observed here, which can be attributed to the progressive accumulation of the NaOH concentration during evaporation.

**Figure 10:**
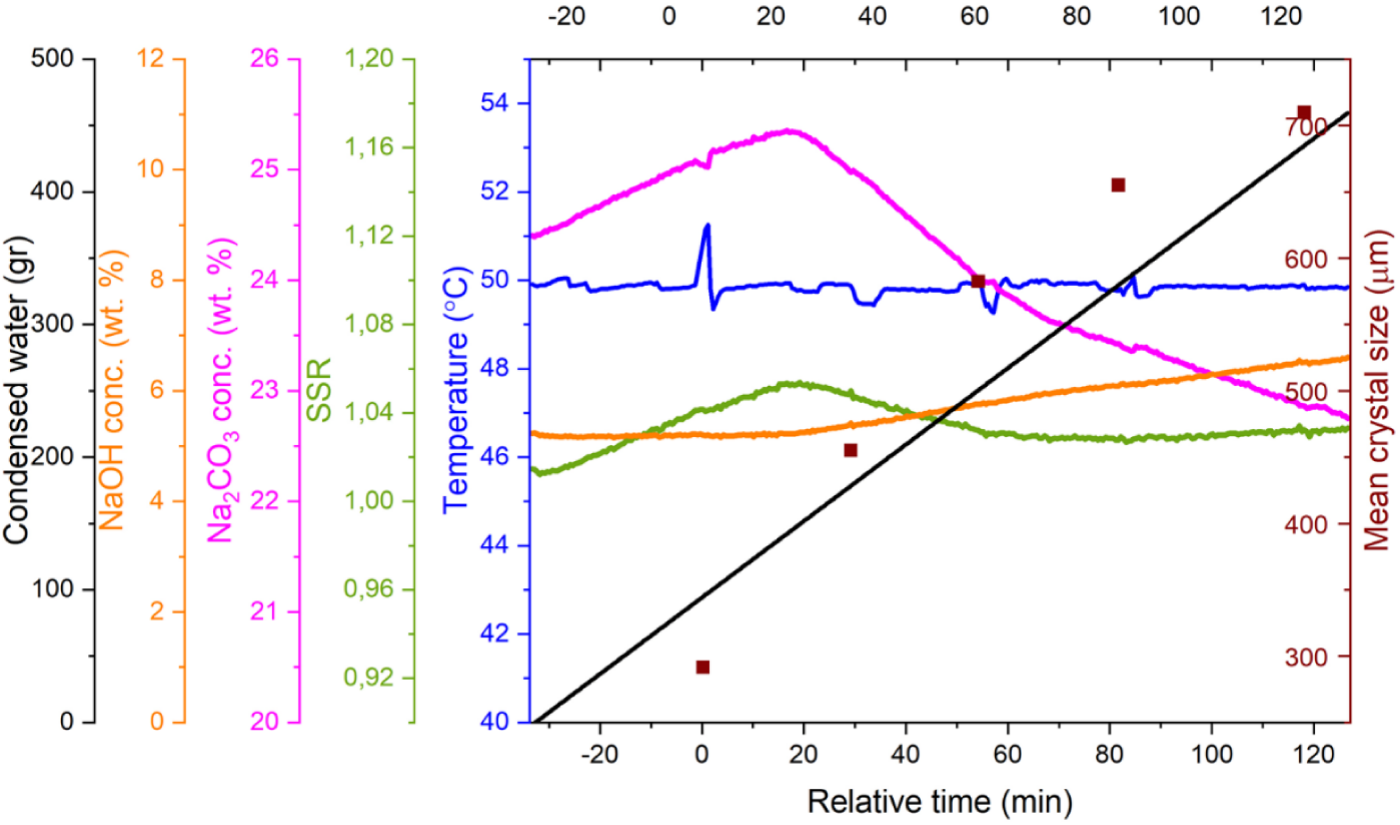
Experimental data from Exp. 5 (*t* = 0 seeding point) including the measured temperature (uncertainty of ±0.05 °C), in-line concentration of Na_2_CO_3_ and NaOH obtained via FTIR, the calculated SSR (relative uncertainty of 2%) measured amount of condensed water (uncertainty of ±2 mg), and offline crystal size measurements conducted using a digital microscope based on minimum length.

**Figure 11:**
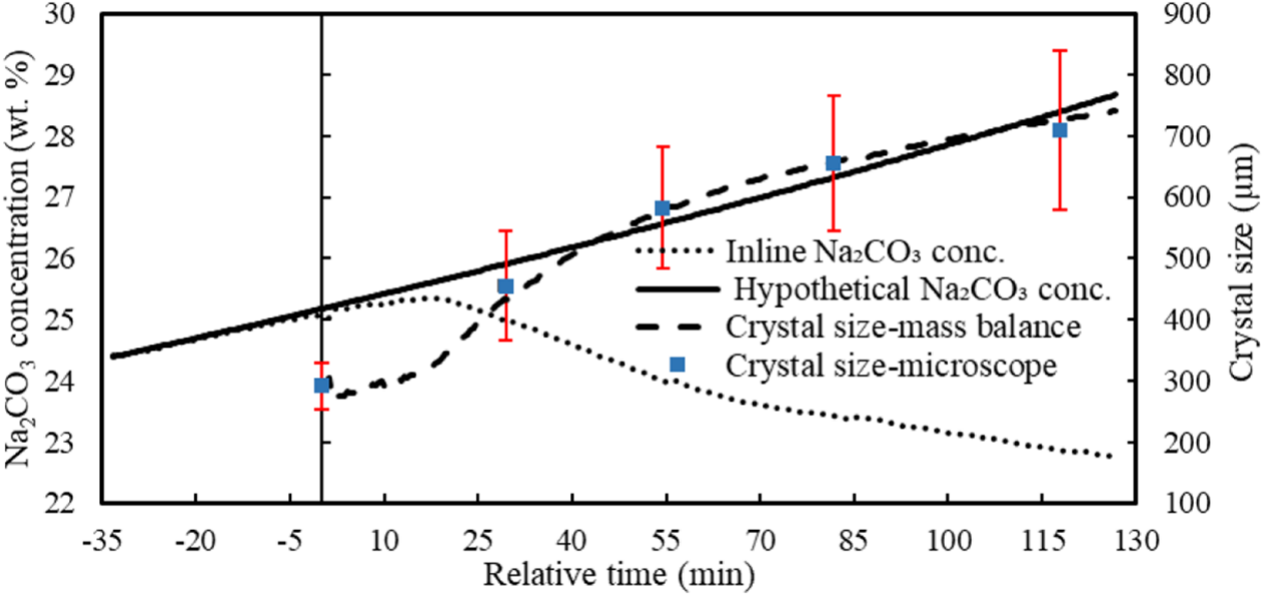
Hypothetical concentration of Na_2_CO_3_ derived solely from evaporation and comparison with the real-time concentration measured in conjunction with the crystal sizes obtained from mass balance and digital microscopy based on the minimum length in Exp. 5 (*t* = 0 seeding point). Red bars represent the standard deviations, the width of the Gaussian distributions for each sample.

Figure 12 depicts the evolution of supersaturation levels alongside crystal sizes measured via microscopy and those predicted by mass balance modeling based on the minimum length for Exps. 5–7. As shown, there is strong agreement between the crystal sizes measured offline and those back-calculated from solution concentrations based on mass balance. This consistency supports the reliability of the applied analytical methods, including FTIR, titration, and collected data from the distillate balance, confirms that crystal growth was the dominant mechanism during the experiments, and reflects the overall quality of the experimental procedures. Exps. 5 and 6 were conducted in the presence of NaOH with varying supersaturation levels by altering seed quantities, Exp. 7 without NaOH to assess its impact on crystal morphology and growth rate. Comparison between Exps. 5 and 7, which shared identical experimental conditions such as evaporation rate, seed quantity, and temperature, revealed faster crystal growth in Exp. 7. This can be attributed to its slightly higher supersaturation and the fact that the absence of NaOH promotes faster growth on the minimum length of the hexagonal face (Table 3). As shown in Figure 13, the presence of NaOH influences crystal habit, as well, thereby affecting the proportionality of crystal dimensions. Table 3 provides these proportions for Na_2_CO_3_·1H_2_O crystal lengths based on the dimensions introduced in Figure 4. The crystal habit depends on the relative growth rates of individual crystal faces, which are strongly influenced by impurities, additives, and solvent molecules. These species can adsorb selectively on specific faces, thereby blocking or slowing their growth. A possible mechanism for the present case is schematically illustrated in Figure 14, which shows the influence of NaOH on crystal habit. In this figure, A_S_ denotes the surface area of a given face, and G_s_ represents its growth rate. The illustration highlights how selective adsorption of NaOH molecules on face 1 reduces its growth rate compared with the other faces. This behavior is supported by experimental data. In the absence of NaOH (Exp. 7), the surface area ratio A_S,1_/A_S,2_ is 1.76, indicating relatively balanced growth between the two faces. However, in the presence of NaOH (Exps. 5 and 6), this ratio significantly increases to 5.44, confirming that growth on face 1 is strongly inhibited, leading to an elongated habit. Measured crystal angles for samples grown with and without NaOH showed no significant variation, indicating that the same crystal faces were preserved during the growth. Just to note, monohydrate crystals tend to form star-shaped agglomerates at high supersaturations composed of radiating elongated subcrystals as shown in the SI, Figure S9.

**Figure 12:**
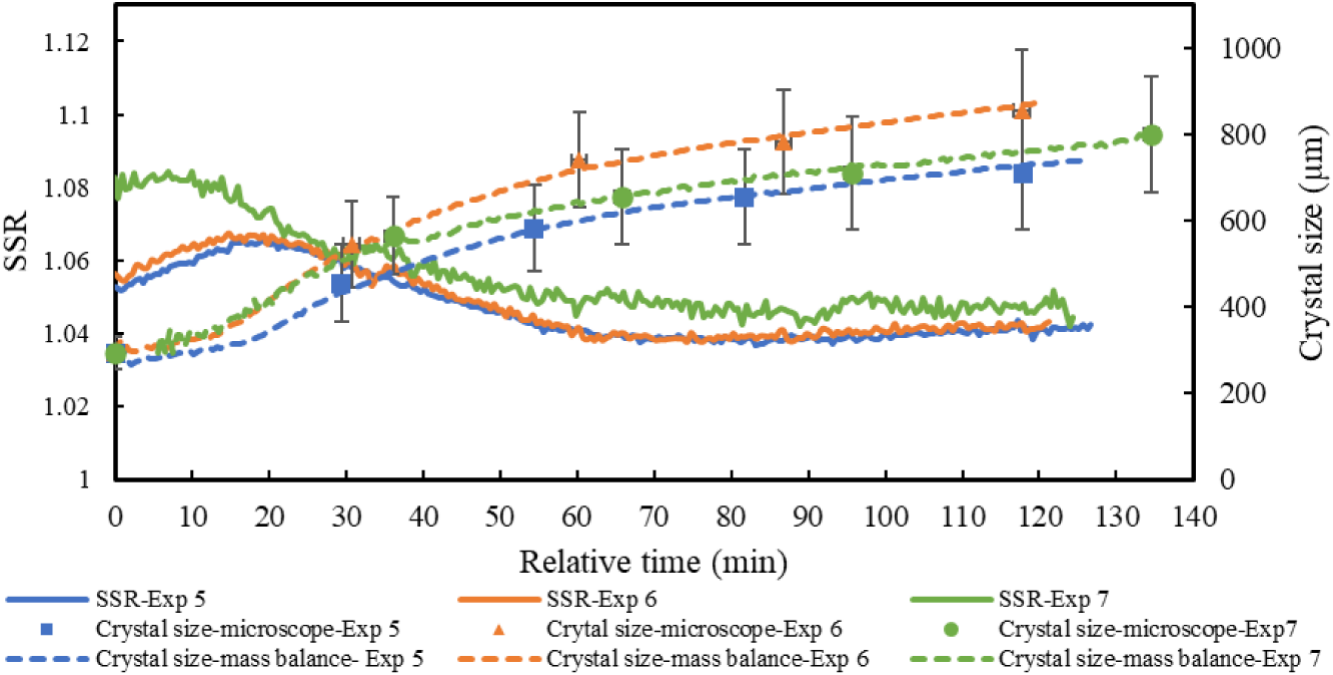
Crystal size evolution based on minimum length and corresponding SSR (relative uncertainty of 2%) (*t* = 0 seeding point) for the vacuum evaporative crystal growth studies of Na_2_CO_3_· 1H_2_O, Exps. 5–7. Bars represent the standard deviations, the width of the Gaussian distributions for each sample.

**Figure 13:**
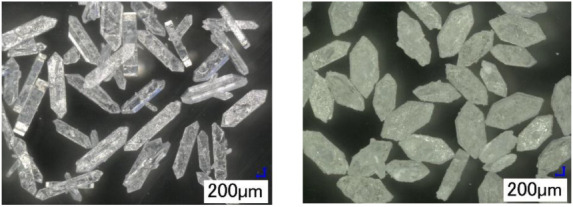
Digital microscope images of Na_2_CO_3_· 1H_2_O crystals obtained in the presence of NaOH, Exp. 5 (left) and without NaOH, Exp. 7 (right).

**Figure 14:**
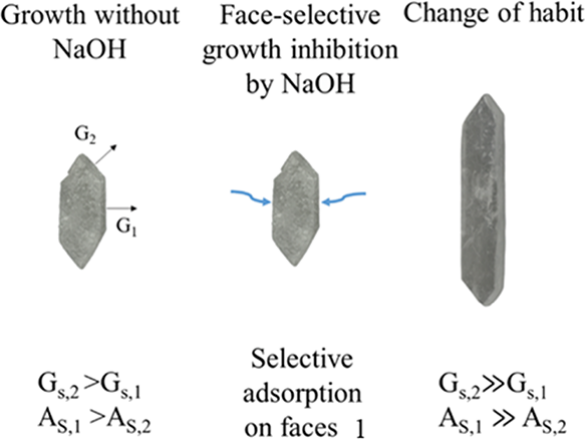
Potential impact of the presence of NaOH on the crystal habit of Na_2_CO_3_·1H_2_O via selective adsorption on crystal faces 1 (with A_S_, surface area and G_s_, surface-area-related growth rate for faces 1 and 2).

The obtained monohydrate crystals were dried at 150 °C to measure the bulk density of the resulting anhydrous form. For instance, sample 3 from Exp. 5, after drying, exhibited an average bulk density of 892 kg/m^3^, which falls within the density range of heavy soda ash. This value reflects the influence of the particle morphology and size distribution.

Since in industry applications monohydrate is turned to anhydrate by drying and being melted with sand for glass-making, the major use of heavy ash, these crystals should have a particle size similar to that of sand so that the two mix easily. In practical terms, when used in glass production, the anhydrous form is preferred, as it eliminates the variability and energy losses associated with the water content in the monohydrate. This results in a more consistent feed behavior, improved thermal efficiency, and better process control during melting. In glass production, the sand used is typically silica sand, which is composed of fine grains of quartz. Glass composes of 60.25% silica sand (SiO_2_), around 19.5% Na_2_CO_3_, 18.1% dolomite (CaMg­(CO_3_)_2_), and some minor constituents as Al_2_O_3_, Na_2_O, K_2_O, and CaO.^30^ In principle, the grain size of the sand influences the melting behavior of glass. The smaller grain size is easier to be melted; however, it could be generated into the small bubble during the dissolution period.^30^ The National Industrial Sand Association^31^ specifies that silica sand used in glass manufacturing should have a grain size between 75 and 1180 μm. It should be noted when Na_2_CO_3_·1H_2_O crystals lose their water and convert into anhydrous, there is a reduction in mass and volume due to the loss of water molecules. Since the monohydrate contains about 14.5% water by weight, the size is expected to decrease with drying. In terms of the volume change, the exact change can vary depending on the crystal structure and packing. The specific volume change would require experimental data or detailed crystallographic analysis to quantify.

### 3.4 Kinetic Parameters for Na_2_CO_3_·10H_2_O and Na_2_CO_3_·1H_2_O Growth

Table 4 contains the obtained kinetic parameters for the experiment conducted in the presence of NaOH. For the monohydrate, since the experiments were conducted at a constant temperature with an average deviation of 0.2 °C, the power-law equation was used instead of the power-law Arrhenius model. The kinetic parameters for Na_2_CO_3_·10H_2_O were determined within a limited range where the growth rate increases with rising supersaturation specifically, in the supersaturation ratio range of 1.000–1.033 and at temperatures between 16.8 °C and 15.3 °C, as shown in SI, Figure S10. Beyond the critical supersaturation ratio of 1.03, the decahydrate tends to exhibit zero-order growth, likely due to the mechanisms discussed in Section 3.2. For the decahydrate the the Arrhenius expression (Eq. 2) was fitted over a very narrow experimental window. Under these conditions, the pre-exponential factor and activation energy become strongly correlated, such that different combinations of comparatively large parameter values yield nearly identical growth rates without affecting the quality of the fit. To evaluate parameter repeatability, 95% confidence intervals were determined for the fitted kinetic parameters. The resulting uncertainty ranges for decahydrate and monohydrate growth are provided in SI, Table S.1. Helfenritter et al.^13^ studied the growth kinetics of a 150 μm film of supersaturated solution in both binary and ternary systems in the presence of Na_2_CO_3_. The results indicated that in the binary system, diffusion and integration limitations were present, with a mass transfer coefficient ranging from 3 × 10^–6^ m s^–1^ < $k_{g,CO_{3}}$ < 14 × 10^–6^ m s^–1^ over a temperature range of 17–23.5 °C and initial supersaturation ratios lower than 1.15 within desupersaturation experiments. The reaction order was found to be 1.4 at 17 °C, according to diffusion–reaction theory. In the ternary system, the kinetics were similar to those in the binary system, with an average reaction order of 1. The obtained parameters for the monohydrate in this work are valid within a supersaturation ratio range of 1.037–1.067 at 50 °C, consistent with the experimental conditions shown in Figure 12. A comparison of the results in Table 4 indicates that the monohydrate has a higher growth order than the decahydrate, which can be attributed to the elevated temperature in the evaporative crystallization enhancing growth kinetics and supporting more favorable crystallization behavior of Na_2_CO_3_·1H_2_O under these conditions.

**Table 4:** Estimated Kinetic Parameters of Crystal Growth for Sodium Carbonate Monohydrate (Based on Minimum Length), and Decahydrate (Based on Maximum Length) in the Na_2_CO_3_–NaOH–H_2_O System (5 wt % NaOH)

Exp. No.	OF_*g*_ ^a^	*k*_*g*,0_ (m/s)	*E*_*A*,*g*_ (J/mol)	*g*
1–2 (decahydrate)	1.2	1.64 × 10^14 9^	8.51 × 10^5^	1.42
5 and 6 (monohydrate)	14.99	9.42 × 10^–5^ ^b^	-	2.35

a Objective function value.

b Amount of *k*_*g*_.

### 3.5 Comparison of Crystal Growth Rate and Yield for Na_2_CO_3_·10H_2_O and Na_2_CO_3_·1H_2_O

In Figure 15a, the modeled crystal growth rates for Na_2_CO_3_ decahydrate and the monohydrate are presented, both based on their maximum observed lengths, denoted as *L*_2_. The growth rates are obtained at constant temperatures of 15.3 °C for the deca- and 50 °C for the monohydrate. The applied supersaturation ranges in each case correspond to those used to obtain the kinetic parameters for the respective hydrate phases. As seen, the decahydrate exhibits lower growth rates, reaching a maximum of around 200 nm/s at a supersaturation of approximately 1.027. Experimental observations indicate that supersaturation levels higher than this point lead to a decline in growth rate, approaching zero-order kinetics. For comparison growth rates of other strongly hydrated inorganic substances reported in ref. 32 can be considered. For example, KAl­(SO_4_)_2_·12H_2_O at 15 °C and a supersaturation ratio of 1.05 shows a growth rate of 22.3 nm/s with a growth order of 1.6. Na_2_HPO_4_·12H_2_O at 20 °C and a supersaturation ratio of 1.05 exhibits a growth rate of 214 nm/s with a growth order of 1.0, while Na_2_SO_4_·10H_2_O at 25 °C and a supersaturation ratio of 1.02 has a growth rate of 189 nm/s with a growth order of 1.0. Therefore, the growth rate obtained for Na_2_CO_3_·10H_2_O falls within the range reported for other hydrated salts.

**Figure 15:**
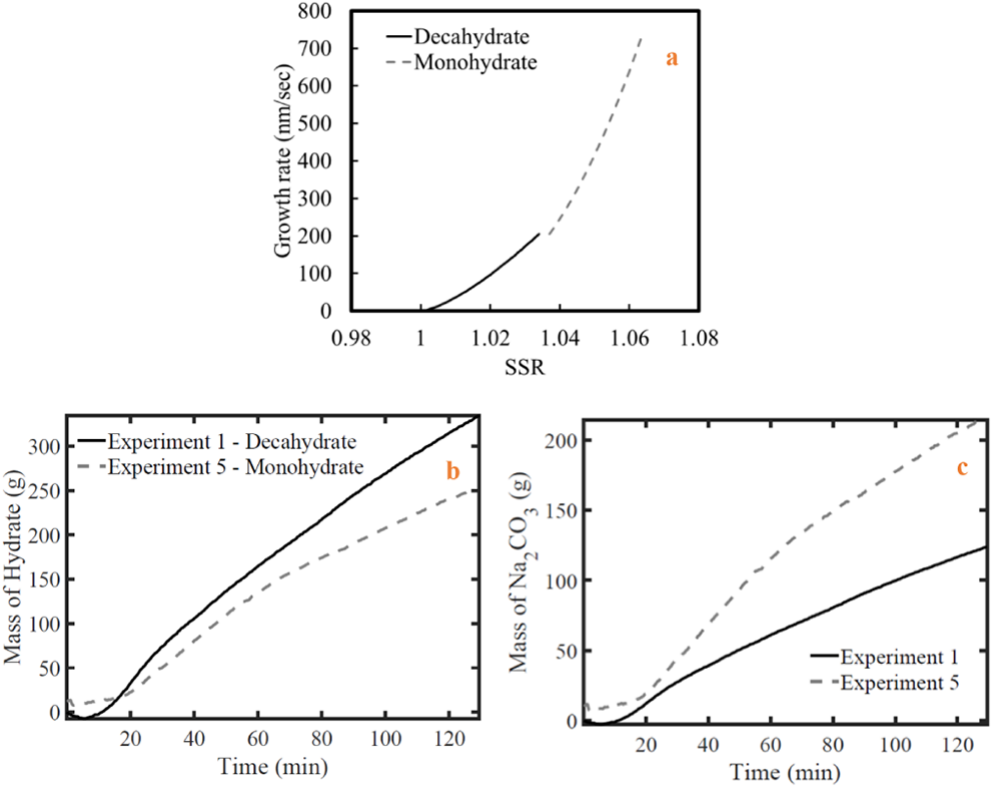
Comparison of Exp. 1 (decahydrate) and Exp. 5 (monohydrate) in terms of a) modeled crystal growth rates along with maximum crystal lengths (*L*_2_); b) produced hydrate mass, and c) produced equivalent anhydrous Na_2_CO_3_ mass in a 3 L-scale crystallizer.

As depicted in Figure 15a, the monohydrate exhibits higher growth rates with values exceeding 700 nm/s at a supersaturation ratio of approximately 1.06. In a research study,^14^ the kinetics of Na_2_CO_3_·1H_2_O was investigated, focusing on the crystal growth during the solution-mediated process where the anhydrous form dissolves, leading to the conversion of Na_2_CO_3_ to Na_2_CO_3_·1H_2_O. Growth rate calculations indicated that monohydrate crystals grow at higher rates at lower aqueous phase temperatures. Growth rates are reported around 1300 and 400 nm/s at supersaturation ratios of 1.25 and 1.075, respectively, and thus in the range determined in this study.

Figure 15b and c provides a comparative overview of the total crystallized product mass, both in hydrate form and in terms of equivalent anhydrous Na_2_CO_3_, for two representative experiments in a 3-L batch crystallizer: **Exp. 1**, the cooling crystallization of the decahydrate at the highest cooling rate, and **Exp. 5**, applying vacuum evaporative crystallization of the monohydrate. The experiments were conducted under a cooling rate of 4.14 K/h and an evaporation rate of 2.88 g/min, respectively. Despite the higher crystal growth rate and density of the monohydrate, Exp. 1 yielded a 31.2% greater accumulated hydrate mass, attributed to the larger volume factor of the decahydrate crystal morphology compared to that of the monohydrate. Additional factors, such as the solubility and the supplied supersaturation rate, also contributed to this outcome. However, when normalized to equivalent anhydrous Na_2_CO_3_, monohydrate crystallization in Exp. 5 produced 76% more soda ash within the same duration (125 min), reflecting its higher Na_2_CO_3_ content per unit mass.

The reduced growth rate of Na_2_CO_3_·10H_2_O at high supersaturation is likely due to its growth mechanism, which limits the yield. Improved growth performance may be achieved at lower supersaturation, where kinetic parameters remain valid. However, further investigation is required to determine whether controlled low-supersaturation crystallization can lead to enhanced yield and product quality in decahydrate production. Given that monohydrate is the final desired product in CODA applications due to its high bulk density, the initial quality of decahydrate crystals may be of limited importance, as they are later redissolved and recrystallized as monohydrate (process variants of P1, P2, and P4).

## 4 Conclusion

This study systematically investigated the crystallization behavior and growth kinetics of sodium carbonate decahydrate (Na_2_CO_3_·10H_2_O) and monohydrate (Na_2_CO_3_·1H_2_O) in the ternary Na_2_CO_3_–NaOH–H_2_O system, with the goal of supporting more sustainable and efficient soda ash production within the CODA process. Cooling crystallization of Na_2_CO_3_·10H_2_O and vacuum evaporative crystallization of Na_2_CO_3_·1H_2_O were conducted in a 3-L batch crystallizer under well-controlled seeding and supersaturation conditions. A validated shortcut method was employed to estimate crystal growth rate parameters under varying temperature and supersaturation conditions. This method enabled efficient parameter estimation by tracking the evolution of crystal sizes derived from mass balance modeling (validated via digital microscopy), supersaturation, and temperature using data from in-line FTIR measurements and solubility models developed in the previous work. Combined with an empirical power-law kinetic model, this approach allowed accurate characterization of growth behavior for each hydrate phase.

The decahydrate exhibited growth kinetics marked by an initial increase in growth rate with rising supersaturation, followed by a decline beyond a critical supersaturation ratio of ∼1.03, ultimately reaching a zero-order growth rate regime. This behavior might be attributed to surface roughening, consistent with the macroscopic morphology analysis. Grown crystals displayed complex internal morphologies, such as hollow cores and sector boundaries, suggesting surface degradation at higher supersaturations. These results indicate that maintaining supersaturation below the critical threshold may facilitate smoother surface growth and improved rates. In batch systems, this can be achieved by adding crystals or reducing the supersaturation through dilution before reaching the critical supersaturation. Near the critical supersaturation, Na_2_CO_3_·10H_2_O growth rates reached ∼200 nm/s in the presence of 5 wt % NaOH. The absence of NaOH had no observable effect on growth behavior or crystal habit.

In contrast, Na_2_CO_3_·1H_2_O demonstrated higher growth rates, and NaOH was found to modify crystal habit via selective adsorption on specific faces and elongating them; hence, crystals were more compact in the absence of NaOH. The onset of nucleation at elevated supersaturation resulted in the formation of star-shaped agglomerates. Kinetic analysis revealed a higher growth order for the monohydrate (*g*  =  2.35) than for the decahydrate (*g* = 1.42), reflecting stronger supersaturation dependence. At a supersaturation ratio of 1.06 at 50 °C, monohydrate growth rates exceeded 700 nm/s in the presence of 5 wt % NaOH. Despite operating at lower final supersaturation values, evaporative crystallization yielded higher equivalent anhydrous Na_2_CO_3_, underscoring the superior efficiency of this method. After drying, the monohydrate crystals produced in this study exhibited bulk densities (892 kg/m^3^), which are within the lower range of that of heavy soda ash, addressing a key requirement for glass manufacturing applications.

From an industrial perspective, the findings emphasize the importance of maintaining supersaturation within optimal ranges to maximize yield and ensure product quality. Future work should focus on in situ diagnostics and scale-up studies to further validate the hypothesized growth phenomena and support the design of robust crystallization processes.

## Supplementary Material



## Notes

Additional details on soda ash production methods, preliminary experiments, seed preparation, FTIR spectroscopy and calibration, and supporting data on crystal growth kinetics (PDF)
