# Supplementary material for: Crystal Growth Kinetics of Na 2 CO 3 Hydrate Phases in the Na 2 CO 3 –NaOH–H 2 O System for Sustainable Soda Ash Production
Source: ACS Omega. 2026 Jan 23;11(5):7741–55. doi: 10.1021/acsomega.5c09440 (PMC12903160; doi:10.1021/acsomega.5c09440)
Supplement: Supplementary file 1 [file ao5c09440_si_001.pdf]

Supplementary Information

Crystal Growth Kinetics of Na_2CO_3 Hydrate Phases in the Na_2CO_3-NaOH-H_2O System for Sustainable Soda Ash Production

Somayyeh Ghaffari *, Jonathan Gänsch, Peter Schulze, and Heike Lorenz

Max Planck Institute for Dynamics of Complex Technical Systems, Magdeburg, 39106 Germany

* ghaffari@mpi-magdeburg.mpg.de

S1. Monohydrate process and Solvay process for soda ash production

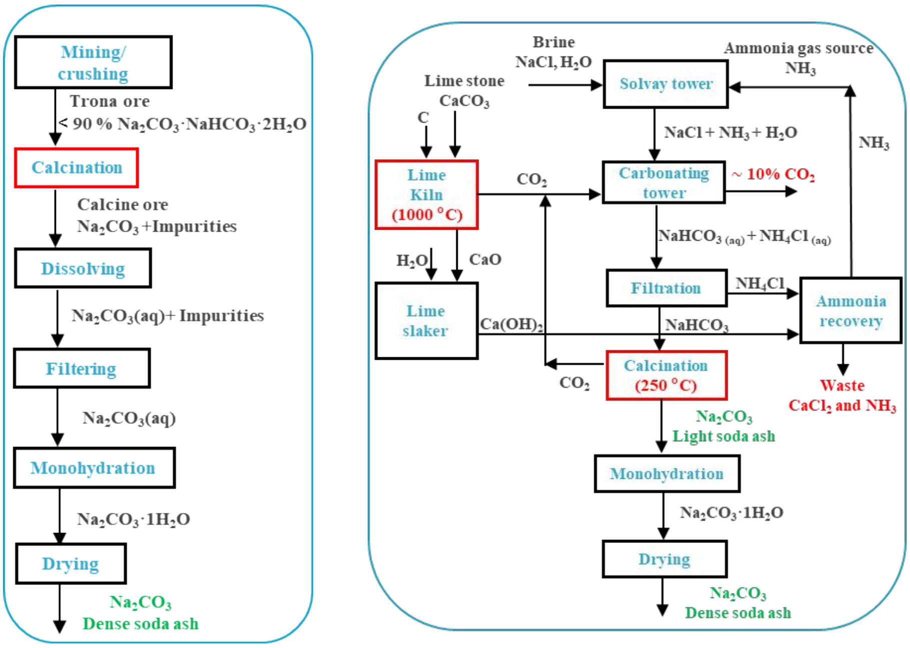

Figure S1. Schematic representation of soda ash production processes via the Trona (Monohydrate) process (left) [3], and the Solvay process (right) [5]. Sub-processes with the highest energy consumption are highlighted in red boxes, while products are indicated in green, and waste materials are marked in red.

S2. Preliminary experiments for $Na_2CO_3·10H_2O$ in the presence of NaOH for seeding strategy

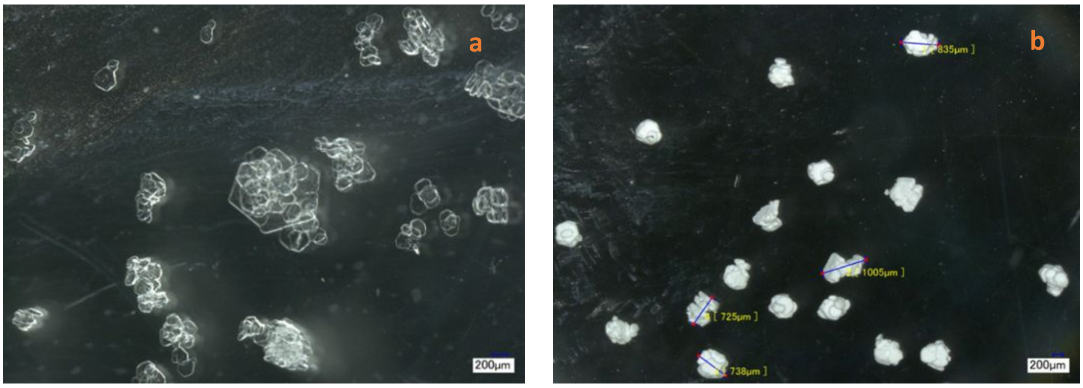

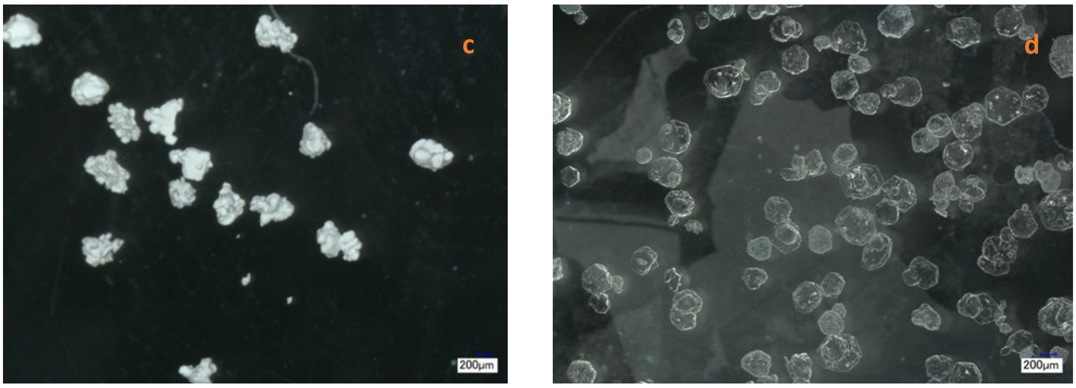

Figure S2. Digital microscope images of the $Na_2CO_3 \cdot 10H_2O$ crystals obtained through cooling experiments in the presence of NaOH with: a)1.45 K subcooling (3rd sample), b) 0.09 K subcooling (5th sample), c) 1.06 K subcooling (5th sample), d) -0.2 K "subcooling" (5th sample) well grown crystals.

S3. Preliminary experiments for $Na_2CO_3 \cdot 10H_2O$ without NaOH for seeding strategy

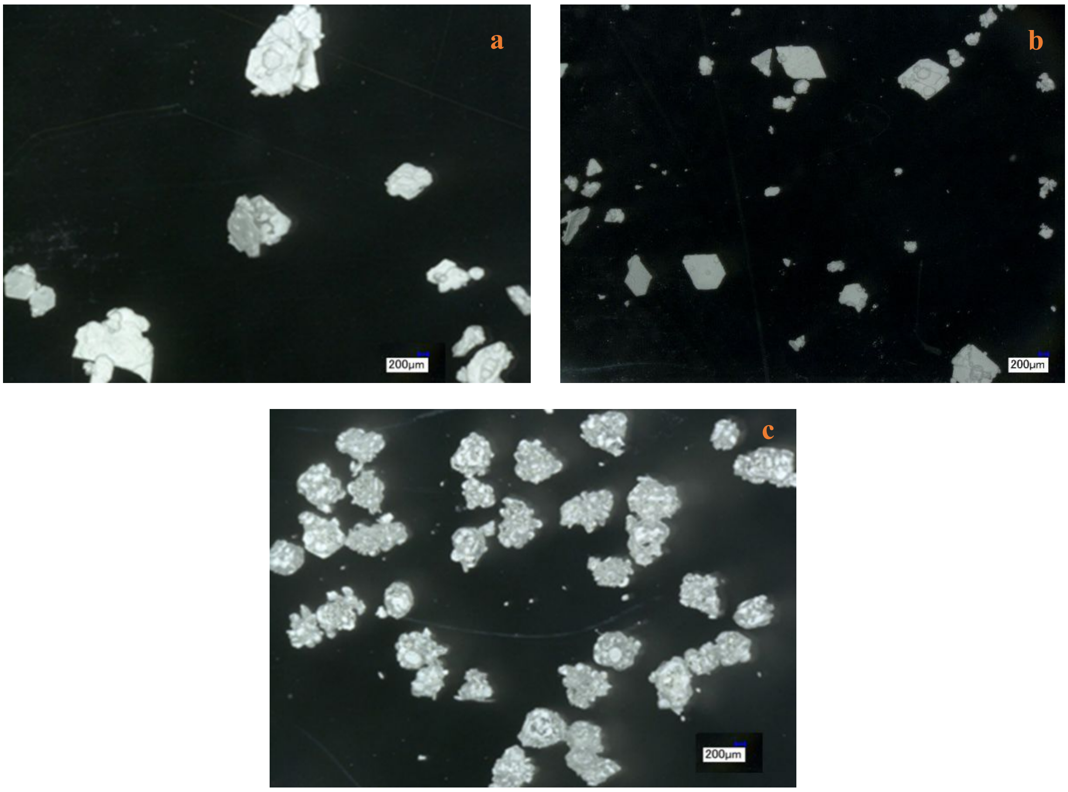

Figure S3. Agglomerated crystals of $Na_2CO_3 \cdot 10H_2O$ formed in cooling experiments under the same conditions as Exp. 4, with subcooling of a) 3.88 K, b) 2.17 K, and c) 0.88 K.

S4. Seed preparation

In Figure S4.1, the vapor pressure of saturated solutions of different salts is compared to the vapor pressure of $Na_2CO_3 \cdot 10H_2O$ crystals, with data based on modified Raoult's law using activity coefficients. The vapor pressure of saturated NaCl solution exceeds that of $Na_2CO_3 \cdot 10H_2O$ crystals, ensuring the rehydration of the decahydrate.
The water content of the hydrates was quantitatively assessed via titration both prior to and following storage in the desiccator. It was observed that the hydration level could increase from 9.3 to 9.5 water molecules over a period of two weeks. It is noteworthy that a newly opened commercial sample of $Na_2CO_3 \cdot 10H_2O$ contained approx. 9.7 water molecules.

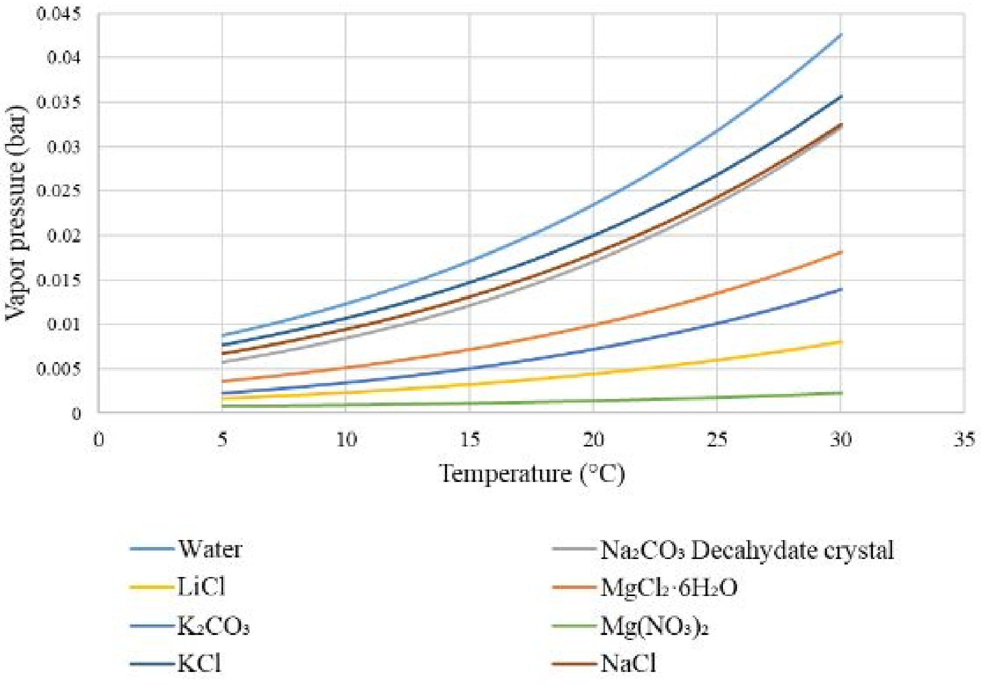

Figure S4.1 Vapor pressure of saturated salt solutions of different salts vs. vapor pressure of solid $Na_2CO_3 \cdot 10H_2O$.

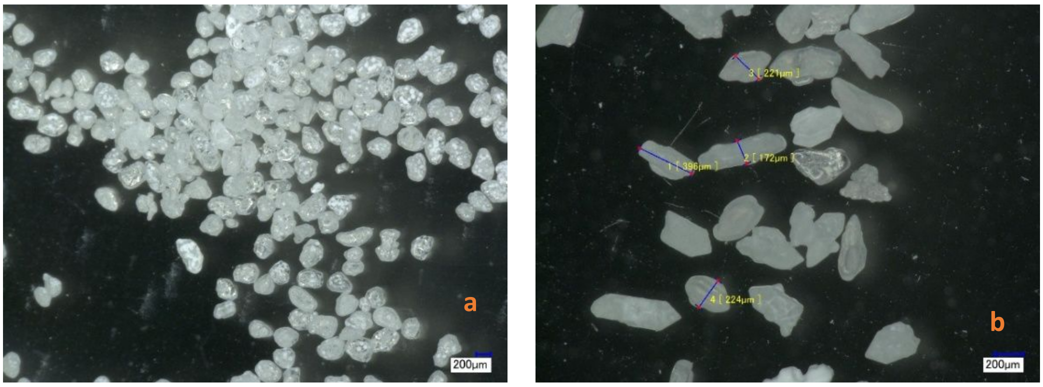

Figure S4.2 Microscope image of seed crystals, a) $Na_2CO_3 \cdot 10H_2O$ and b) $Na_2CO_3 \cdot 1H_2O$ obtained from sieving with a size fraction of 212-250 µm.

S5. FTIR Spectroscopy and Calibration Methodology

Figure S5.1 shows two examples of the FTIR spectra of aqueous mixtures of Na_2CO_3 and NaOH at varying concentrations and temperatures. In the FTIR spectra, key absorbance bands appear at 1637 cm^{-1} (H_2O bending vibration) and 1386 cm^{-1} (CO_3^{2-} stretching). At constant concentration, decreasing the temperature increases the intensity at wavenumber 1386 cm^{-1}, while reducing the temperature decreases the intensity at wavenumber 1637 cm^{-1}. At constant temperature, an increase in Na_2CO_3 concentration enhances the intensity at wavenumber 1386 cm^{-1}. The effect of concentration is greater than the effect of temperature on this wavelength. The effect of temperature on 1386 cm^{-1} is more significant than on other wavelength ranges. NaOH has an indirect effect on the spectra; increasing NaOH concentration shifts the entire spectrum upwards. Incorporating these effects into the calibration process introduces complexity, thereby necessitating the utilization of process samples for calibration. Given that all experiments were conducted within consistent temperature ranges for each hydrate, offline measurements from all experiments were integrated with additional independent calibration samples. These additional samples had their spectra recorded at a constant concentration across varying temperatures to establish a robust calibration model. A total of 72 samples were employed to develop the calibration for cooling crystallization experiments. Since the vacuum evaporative crystallization experiments were conducted at constant temperatures, only process samples were utilized for calibration. The FTIR probe employed was also capable of inline temperature measurement, allowing for the consideration of temperature variations in the calibration. The iC Quant package on iC.IR 4.3.35 software was utilized for the calibration. The iC Quant modeling package is designed for model construction that can subsequently be applied for predictions. Data for the modeling package was obtained from the iC experiments, and the calibration method used was PLS (Partial Least Squares) multivariate analysis. The data is organized as process variables/predictors and response data. The variables included temperature and the entire spectra, while the response data comprised offline measurements of NaOH and Na_2CO_3 concentrations. The temperature measurements of the probe were calibrated using ice and boiled water; however, there was still a deviation from the temperature measured by the Pt100 sensor. Consequently, a new temperature trend was introduced to the software. After specifying the predictor and response data, the user can calibrate and validate the model. IC Quant calculates the model parameters and displays the optimal factor if the Auto-select factors checkbox is checked. The optimal number of factors is determined based on the minimum in PRESS plots. The PRESS vs. Factor plot is a diagnostic tool used in statistical modeling, particularly in regression analysis, to assess a model's predictive capability. PRESS stands for "Predicted Residual Error Sum of Squares," which measures how well a regression model predicts new data. A lower PRESS value indicates a superior predictive model. By examining this plot, one can determine the optimal number of factors to include in the model. The objective is to identify a point where adding more factors does not significantly decrease the PRESS value, indicating that additional factors do not enhance the model's predictive power, thus avoiding overfitting. The factors used for both NaOH and Na_2CO_3 concentrations were three. Once the model is fitted to experimental data, it can be used for concentration prediction every 30 seconds. Figure S5.2 illustrates the alignment between off- and inline measurements for a representative cooling and vacuum evaporative crystallization experiment. The coefficients of determination (R^2) were 0.99 and 0.98, for Na_2CO_3 and NaOH, respectively.

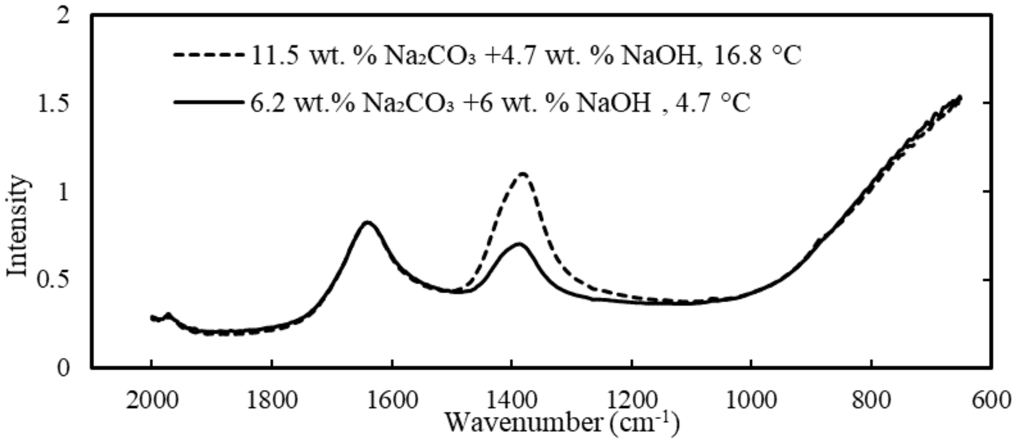

Figure S5.1. FTIR spectra of aqueous mixtures of Na_2CO_3 and NaOH at varying concentrations and temperatures.

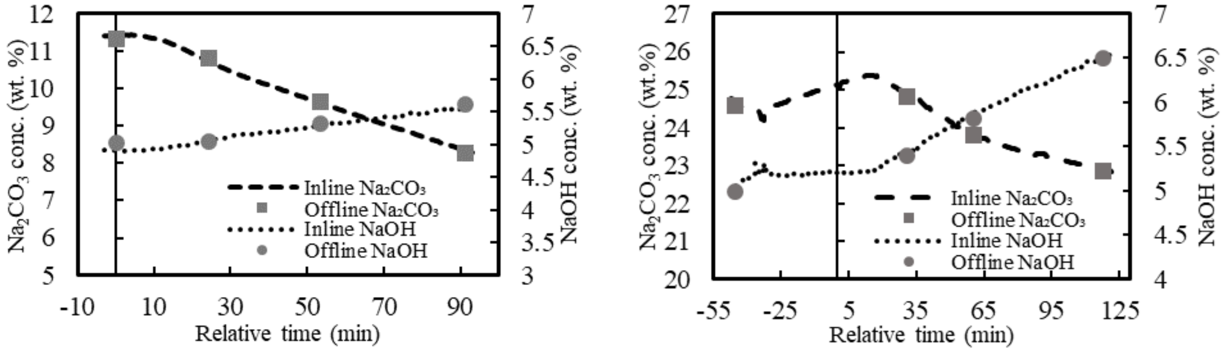

Figure S5.2. In- and offline concentration measurements (t=0: seeding point) in the system of Na_2CO_3, NaOH and H_2O within a cooling crystallization experiment, Exp. 1 (left), and a vacuum evaporative crystallization run, Exp. 6 (right). The coefficients of determination (R^2) were 0.99 and 0.98, for Na_2CO_3 and NaOH, respectively.

S6. Solid phase analysis: Single crystal

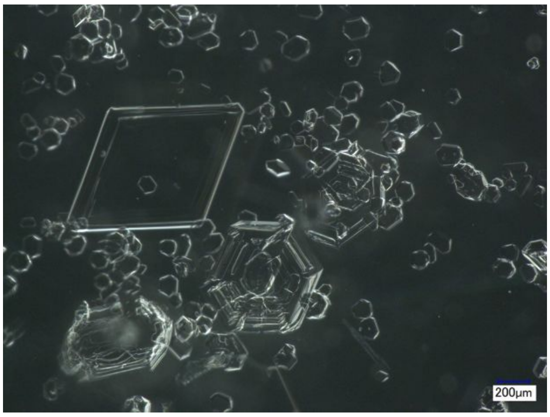

Figure S6. Digital microscope image of single crystals of $Na_2CO_3{\cdot}10H_2O$ grown at room temperature in the presence of 5 wt. % NaOH through slow evaporation.

S7. Crystal size evolution obtained by mass balance

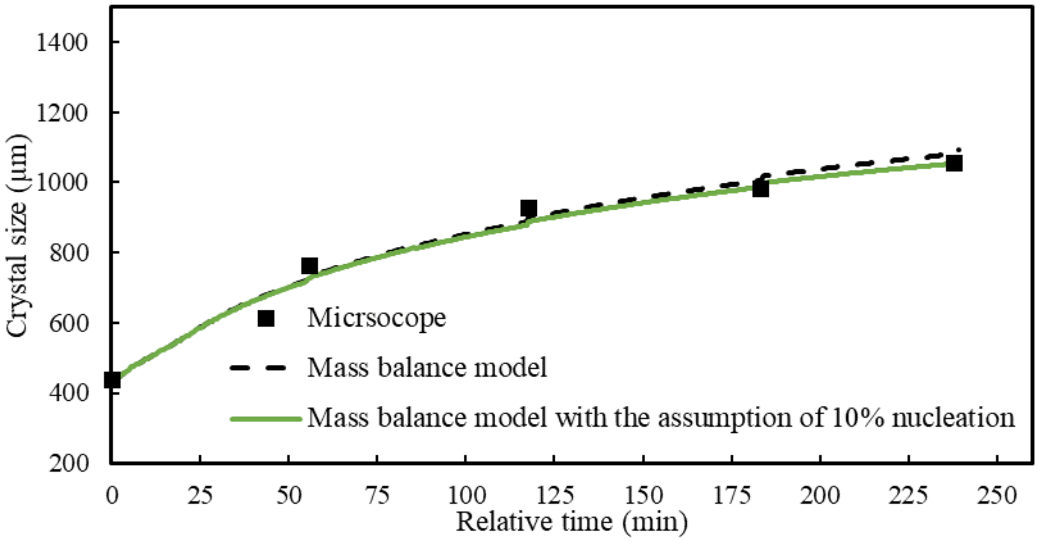

Figure S7. Crystal size model for Exp. 2 based on the inline concentration measurements, with the assumption of nucleation.

S8. Growth rate of $Na_2CO_3{\cdot}10H_2O$ crystals

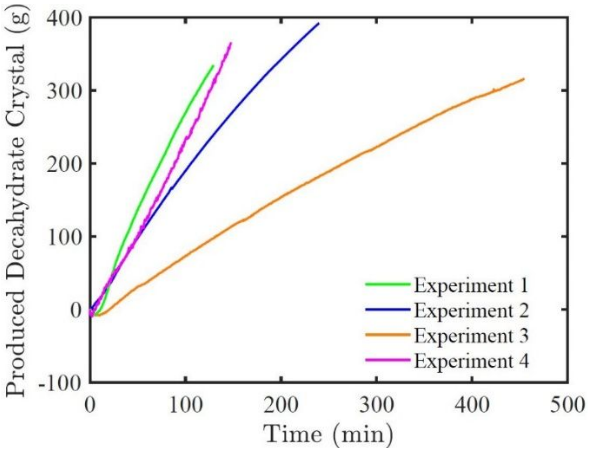

Figure S8. Produced amount of $Na_2CO_3{\cdot}10H_2O$ crystals (t=0 seeding point) for Exps. 1-4.

S9. $Na_2CO_3{\cdot}1H_2O$ crystals in the case of agglomeration

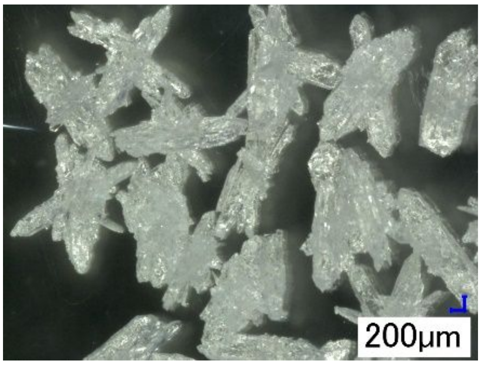

Figure S9. Star-shape agglomerates of $Na_2CO_3{\cdot}1H_2O$ crystals.

S10. Kinetic parameters for $Na_2CO_3 \cdot 10H_2O$

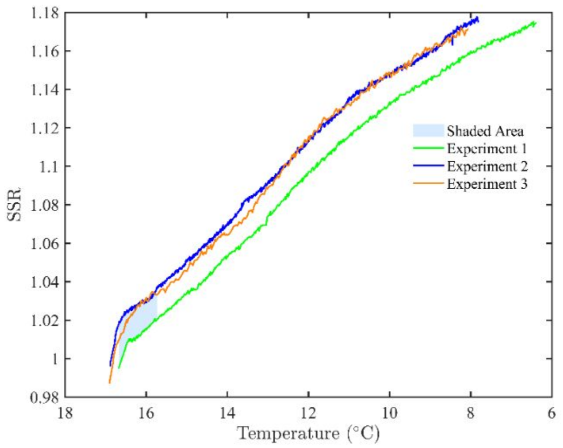

Figure S10. Temperature and supersaturation ratio range (shaded area) within which the kinetic parameters for $Na_2CO_3 \cdot 10H_2O$ were determined.

In Table S1, the 95% confidence intervals of the fitted growth kinetic parameters for both hydrate phases were determined from the covariance matrix of the nonlinear least-squares regression using the Jacobian of the objective function evaluated at the optimum.

Table S1. 95% Confidence Intervals for growth kinetic parameters of Na_2CO_3 hydrate phases.

	log ($K_{g,0}$)	$E_{A,g}$	g
Decahydrate	[35.2 – 263.3]	[2.07×10^5 –1.49×10^6]	[0.49-2.35]
Monohydrate	[−4.62 – −3.43]	-	[1.88–2.82]
